# SERPINB7 maintains skin barrier by regulating protein O-GalNAc glycosylation

**DOI:** 10.1038/s41420-025-02935-6

**Published:** 2025-12-30

**Authors:** Rui Ma, Chen Peng, Wenjuan Chen, Yizhen Zhang, Yangfeng Ding, Xin Wang, Yuling Shi

**Affiliations:** 1 Department of Dermatology, Shanghai Skin Disease Hospital, Tongji University School of Medicine, Shanghai, China; 2 Institute of Psoriasis, Tongji University School of Medicine, Shanghai, China; 3 Psoriasis Clinical Research Center, Tongji University, Shanghai, China

**Keywords:** Skin diseases, Glycosylation

## Abstract

The skin barrier is crucial for protecting against environmental challenges, preventing water loss, and regulating immune responses. This study aims to investigate the roles and mechanisms of SERPINB7 in skin barrier maintenance. We found that SERPINB7 deficiency disrupts tight junctions of keratinocytes in vitro, and specific knockout of *Serpinb7* in keratinocytes impairs skin barrier function in vivo. SERPINB7 deficiency leads to reduced expression of O-GalNAc regulatory proteins and structural abnormalities in the Golgi apparatus, ultimately impairing protein O-GalNAc glycosylation. Legumain acts as a critical mediator in the maintenance of normal biological functions and O-GalNAc glycosylation regulated by SERPINB7. O-GalNAc inhibition exhibits biological effects analogous to those induced by SERPINB7 deficiency, leading to weakened tight junctions, reduced cell adhesion, and compromised skin barrier integrity in keratinocytes and mouse skin, respectively. Consequently, O-GalNAc deficiency exacerbates inflammatory skin diseases such as psoriasis and atopic dermatitis. Mechanistically, O-GalNAc deficiency primarily affects the glycosylation of calcium-related and cell adhesion-related proteins, disrupting calcium signaling and compromising cell adhesion, ultimately leading to skin barrier dysfunction. In summary, this study demonstrates that SERPINB7 maintains skin barrier through protein O-GalNAc glycosylation. These findings not only deepen our understanding of skin barrier biology but also provide new insights for developing therapeutic strategies for skin barrier-related diseases.

## Introduction

Human skin serves as a protective barrier against the external environment, effectively preventing water loss, excluding toxins, resisting mechanical stress, and participating in immune responses [1]. Many skin diseases, such as atopic dermatitis (AD) and psoriasis, are closely associated with disruptions in the skin barrier [2, 3]. Keratinocytes, the primary cells of the epidermis, play a pivotal role in the establishment and maintenance of the skin barrier through their normal physiological functions, including differentiation, adhesion, tight junctions, metabolism, and immune regulation [4, 5]. Therefore, identifying factors that affect keratinocytes biology and, consequently, impair skin barrier function provides valuable insights for the treatment of skin barrier-related diseases.

Previous research has established a link between the deficiency of SERPINB7, a member of the serine protease inhibitor (serpin) superfamily, and skin barrier disruption [6, 7]. SERPINB7 is abundantly expressed in keratinocytes, with particularly high levels in the stratum corneum and stratum granulosum of the epidermis. Studies have shown that SERPINB7 promotes keratinocyte differentiation and dampens inflammation in psoriasis in a calcium-dependent manner [6]. Furthermore, SERPINB7 has been identified as a cysteine protease inhibitor, with Legumain pinpointed as a key target [7]. The overactivation of Legumain due to SERPINB7 deficiency has been implicated in the impaired skin barrier seen in Nagashima-type palmoplantar keratoderma [7]. Despite these findings, many questions persist regarding the influence of SERPINB7 on the skin barrier. For example, it remains unclear whether skin barrier disruption in animal models mediated by SERPINB7 deficiency is keratinocyte-dependent. Furthermore, the intricate mechanism responsible for the calcium-dependency of SERPINB7-mediated biological effects, as well as the comprehensive understanding of the roles that SERPINB7 plays in influencing the skin barrier, remain incompletely elucidated.

Therefore, based on existing research, we conducted an in-depth exploration of the functions of SERPINB7 in skin keratinocytes. Given the protease inhibitory function of SERPINB7, we employed proteomic analysis to further investigate its downstream effects. We identified protein O-GalNAc glycosylation as a downstream target of SERPINB7 and focused our investigation on the impact of this modification on the biology of keratinocytes and the skin barrier.

## Results

### SERPINB7 deficiency disrupts keratinocyte tight junctions and skin barrier function

**Fig. 1:**
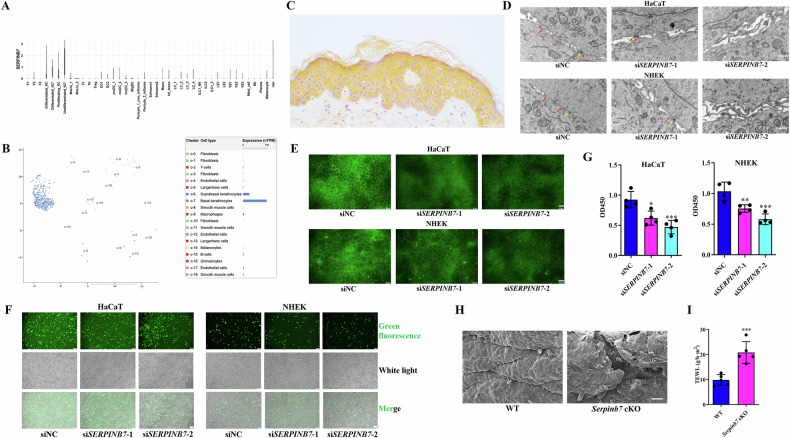
SERPINB7 deficiency disrupts keratinocyte tight junctions and skin barrier function. **A** Relative mRNA expression of *SERPINB7* across different skin cell types analyzed from a single-cell transcriptome profiling (E-MTAB-8142). **B** Relative mRNA expression of *SERPINB7* in various skin cell types obtained from the Human Protein Atlas database (https://www.proteinatlas.org/). **C** Immunohistochemical staining showing SERPINB7 protein expression in human normal skin tissue. SERPINB7 was knocked-down by siRNAs in keratinocytes: **D** Transmission electron microscopy images showing alterations in tight junctions between keratinocytes (red arrow: tight junction; yellow arrow: desmosome; scale bar: 1 μm); **E** Immunofluorescence staining showing changes in the expression of the tight junction protein Occludin (scale bar: 25 μm); Cell adhesion assay evaluating cell-to-cell (**F**) and cell-to-matrix adhesion (**G**) ability of keratinocytes (scale bar: 100 μm). *Serpinb7* was conditionally knocked-out in keratinocytes of C57BL/6 mice: **H** Scanning electron microscopy images revealing changes in the mouse epidermis; **I** Measurement of transepidermal water loss to assess skin barrier function. ^*^P < 0.05, ^**^P < 0.01, ^***^P < 0.001.

To elucidate the functional roles of SERPINB7 in human skin, we analyzed its mRNA and protein expression profiles. Our findings, derived from single-cell transcriptome analysis, database interrogation, and immunohistochemical staining, consistently demonstrated that *SERPINB7* mRNA and protein are predominantly expressed in keratinocytes (Fig. 1A–C). To further explore the functions of SERPINB7 in keratinocytes, we knocked down SERPINB7 in HaCaT and Primary neonatal human epidermal keratinocyte (NHEK) cells (Supplementary Fig. 1). We observed a significant reduction in tight junctions between keratinocytes, accompanied by decreased expression of the tight junction protein Occludin following *SERPINB7* silencing (Fig. 1D, E). Furthermore, both cell-to-cell and cell-to-matrix adhesion were compromised after *SERPINB7* silencing (Fig. 1F, G).

To translate these findings into a more physiologically relevant context, we generated mouse models with specific knockout of *Serpinb7* in keratinocytes. These mice exhibited cracks and fissures (Fig. 1H) and increased Trans Epidermal Water Loss (TEWL) (Fig. 1I) in the dorsal skin, which indicates impaired skin barrier. Taken together, these results suggest that SERPINB7 deficiency impairs keratinocyte adhesion and disrupts tight junctions in vitro, while in vivo, it compromises skin barrier integrity in a keratinocyte-dependent manner.

### SERPINB7 is essential for protein O-GalNAc modification

**Fig. 2:**
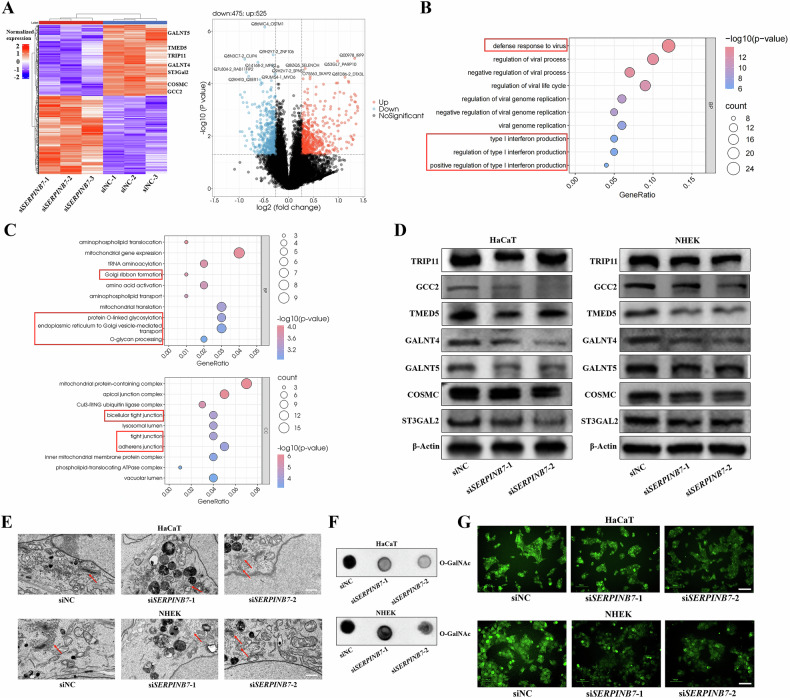
SERPINB7 is essential for protein O-GalNAc modification. **A** TMT-based proteomic analysis revealing differentially expressed proteins following SERPINB7 knockdown. Gene Ontology (GO) enrichment analysis of upregulated (**B**) and downregulated (**C**) proteins of the proteomic analysis. SERPINB7 was knocked-down by siRNAs in keratinocytes: **D** Western blot assay validating the downregulation of Golgi structure and function-related proteins and O-GalNAc glycosylation-related proteins identified in the proteomic analysis; **E** Transmission electron microscopy images showing structural changes in the Golgi apparatus (scale bar: 1 μm); Analysis of O-GalNAc glycosylation changes using dot blot assay (**F**) and an O-GalNAc Modified Glycoprotein Assay Kit (**G**) (scale bar: 100 μm).

We then investigated the molecular mechanisms underlying the disruption of keratinocyte tight junctions and skin barrier integrity caused by SERPINB7 deficiency. Given the role of SERPINB7 as a cystatin-like serine protease inhibitor, we performed Tandem mass tag (TMT)-based proteomic analysis following SERPINB7 knockdown. This analysis revealed many abnormally expressed proteins (Fig. 2A). Through pathway and functional enrichment analyzes, we found that upregulated proteins were enriched in antiviral immune and interferon signaling pathways (Fig. 2B, and Supplementary Fig. 2), indicating that silencing *SERPINB7* enhances immune and inflammatory responses in keratinocytes. Additionally, downregulated proteins were enriched in Golgi structure and function, and in protein O-GalNAc glycosylation, as well as in tight junction and cell adhesion (Fig. 2C, and Supplementary Fig. 3). The decrease in proteins related to tight junction and cell adhesion can partially account for the impaired cell adhesion and tight junction following *SERPINB7* silencing. Furthermore, given that key enzymes mediating O-GalNAc glycosylation and the modification process itself are predominantly localized to and function within the Golgi apparatus, we hypothesized that SERPINB7 deficiency might disrupt the Golgi apparatus and impair protein O-GalNAc glycosylation.

Subsequently, we validated the proteomic findings in HaCaT and NHEK cells, confirming altered expression of Golgi apparatus structure and function-related proteins such as TRIP11, GCC2, and TMED5 (Fig. 2D), as well as O-GalNAc glycosylation-related proteins including GALNT4, GALNT5, COSMC, and ST3GAL2 (Fig. 2D). Moreover, transmission electron microscopy of keratinocytes after *SERPINB7* silencing revealed changes in the Golgi apparatus, characterized by luminal atrophy, structural disorder, and loss of polarity (Fig. 2E). Finally, we confirmed that silencing *SERPINB7* downregulates the level of O-GalNAc glycosylation (Fig. 2F, G). Collectively, these results demonstrate that SERPINB7 is crucial for protein O-GalNAc glycosylation modification.

### Legumain mediates alterations in O-GalNAc, tight junction disruption, and skin barrier damage due to SERPINB7 deficiency

**Fig. 3:**
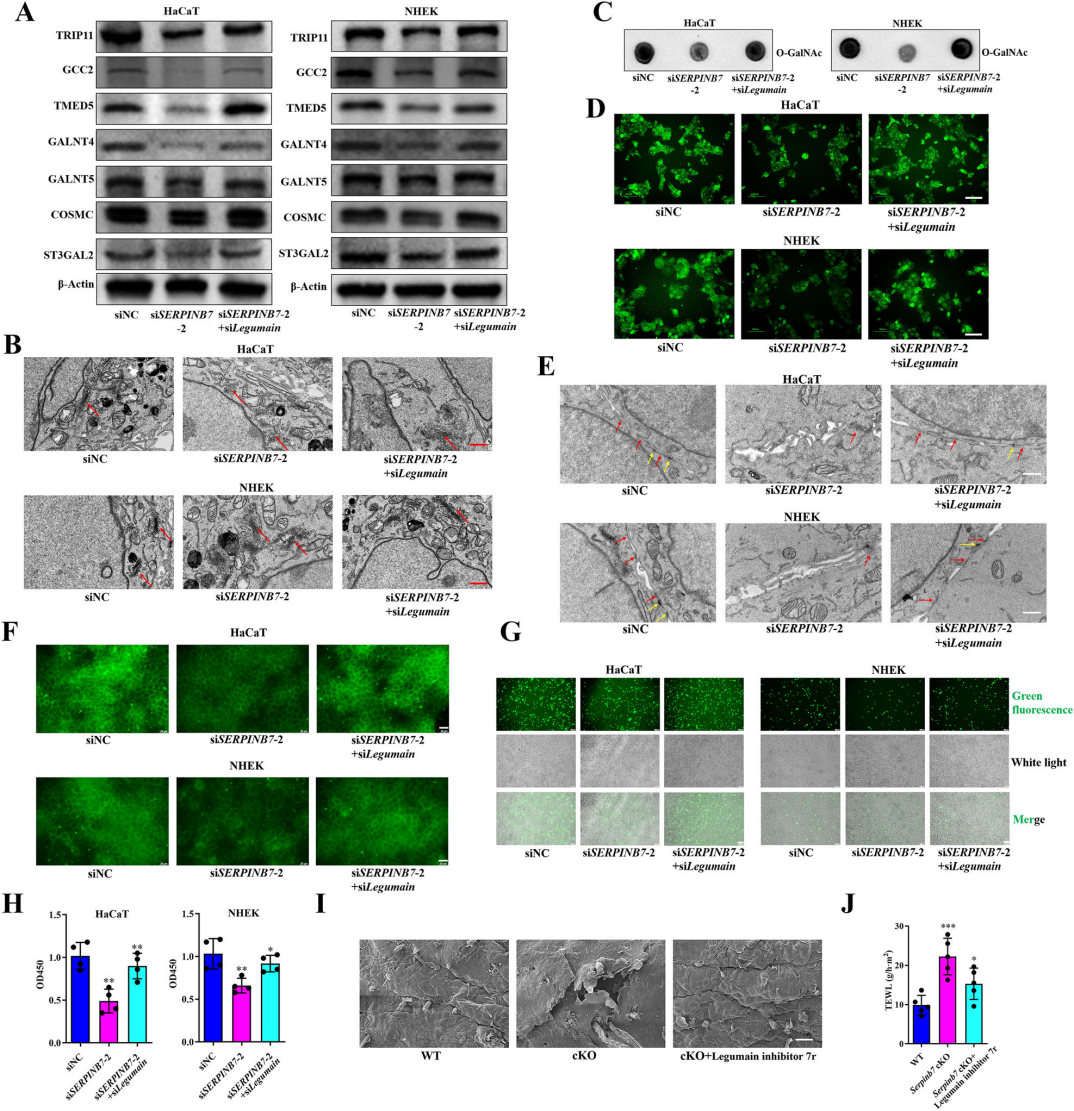
Legumain mediates alterations in O-GalNAc, tight junction disruption, and skin barrier damage due to SERPINB7 deficiency. Keratinocytes were transfected with *SERPINB7* siRNAs alone or together with *Legumain* siRNAs: **A** Western blot analysis evaluating the effects on Golgi structure and function-related proteins and O-GalNAc glycosylation-related proteins; **B** Transmission electron microscopy images showing changes in the Golgi apparatus; Analysis of O-GalNAc glycosylation changes using dot blot assay (**C**) and an O-GalNAc Modified Glycoprotein Assay Kit (**D**); **E** Transmission electron microscopy images depicting alterations in tight junctions between keratinocytes (red arrow: tight junction; yellow arrow: desmosome; scale bar: 1 μm); **F** Immunofluorescence staining showing changes in the expression of the tight junction protein Occludin (scale bar: 25 μm); Cell adhesion assays assessing cell-to-cell (**G**) and cell-to-matrix adhesion (**H**) of keratinocytes (scale bar: 100 μm). Legumain inhibitor 7r was topically applied on the skin of keratinocytes *Serpinb7* cKO mice: **I** Scanning electron microscopy images revealing changes in the mouse epidermis; **J** Measurement of transepidermal water loss to evaluate skin barrier function. ^*^P < 0.05, ^**^P < 0.01, ^***^P < 0.001.

A previous study has demonstrated that SERPINB7 possesses cysteine protease inhibitory properties and has identified Legumain as a key target protease for SERPINB7, and SERPINB7 inhibits Legumain in a protease-substrate manner [7]. To investigate whether Legumain plays a pivotal role in the alterations of O-GalNAc glycosylation observed in SERPINB7 deficiency, we assessed the impact of silencing Legumain on the expression changes of downstream proteins mediated by SERPINB7 deficiency. Our findings revealed that the knockdown of Legumain reversed the downregulation of Golgi structure and function-related proteins, including TRIP11, GCC2, and TMED5, as well as O-GalNAc glycosylation-associated proteins such as GALNT4, GALNT5, COSMC, and ST3GAL2, which were mediated by SERPINB7 deficiency (Fig. 3A). Similarly, the silencing of Legumain also reversed the Golgi abnormalities (Fig. 3B) and alterations in O-GalNAc glycosylation (Fig. 3C, D) induced by SERPINB7 deficiency. Furthermore, we confirmed that the knockdown of Legumain rescued the disruption of tight junctions (Fig. 3E, F) and the reduction in cell adhesion (Fig. 3G, H) caused by *SERPINB7* silencing. In mice with *Serpinb7* cKO in keratinocytes, we observed that Legumain inhibitors reversed the skin barrier disruption caused by *Serpinb7* knockout (Fig. 3I, J). These findings collectively suggest that Legumain mediates the alterations in O-GalNAc glycosylation, tight junction disruption, and skin barrier damage that occur due to SERPINB7 deficiency.

### O-GalNAc inhibition influences keratinocytes tight junction and skin barrier

**Fig. 4:**
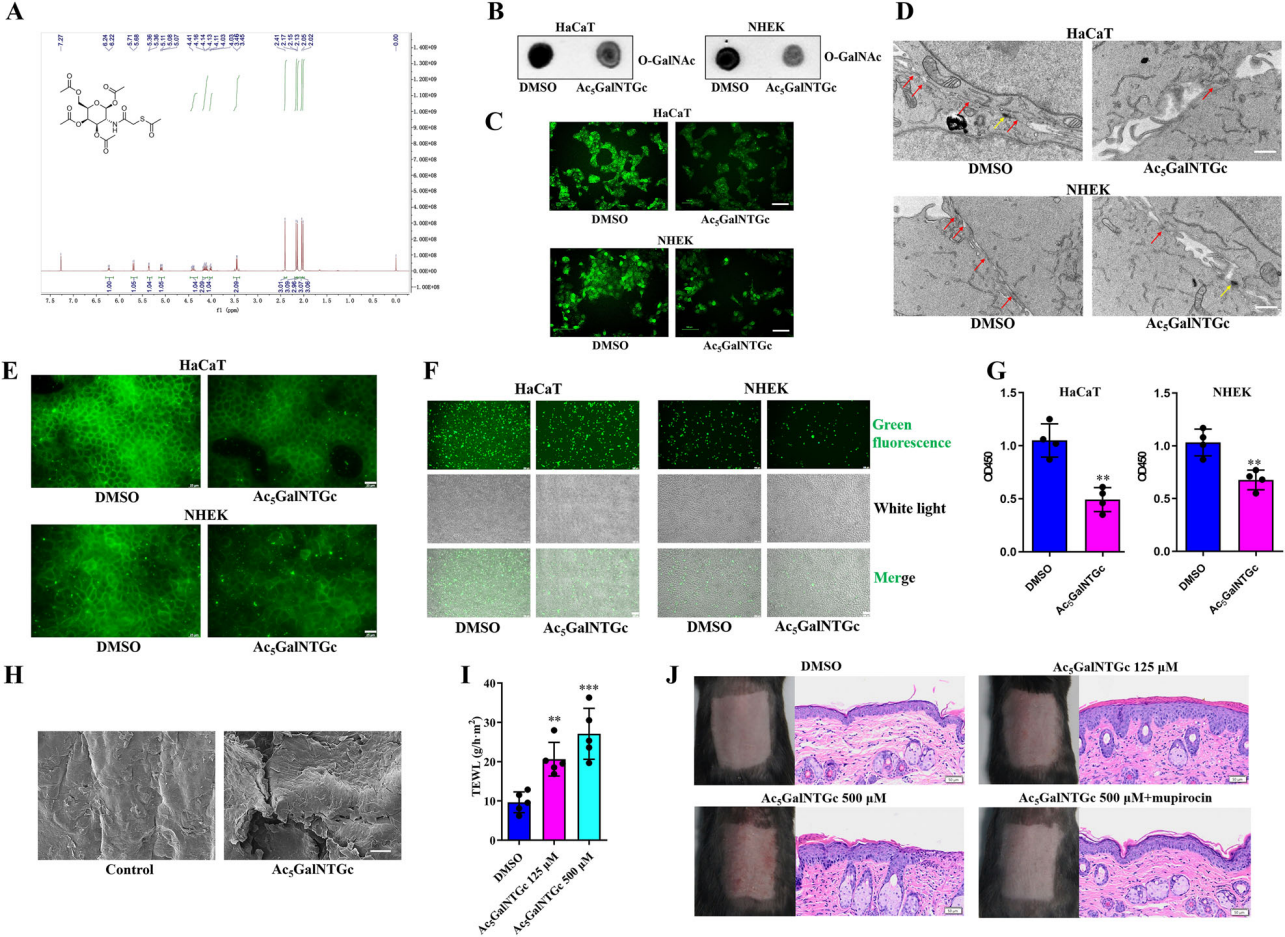
O-GalNAc inhibition influences keratinocytes tight junction and skin barrier. **A** Verification of Ac_5_GalNTGc by Nuclear Magnetic Resonance Spectroscopy of Hydrogen and Mass Spectrometry. Keratinocytes were treated with Ac_5_GalNTGc: Analysis of O-GalNAc glycosylation changes using dot blot assay (**B**) and an O-GalNAc Modified Glycoprotein Assay Kit (**C**) (scale bar: 100 μm); **D** Transmission electron microscopy images showing alterations in tight junctions between keratinocytes (red arrow: tight junction; yellow arrow: desmosome; scale bar: 1 μm); **E** Immunofluorescence staining demonstrating changes in the expression of the tight junction protein Occludin (scale bar: 25 μm); Cell adhesion assays evaluating cell-to-cell (**F**) and cell-to-matrix adhesion (**G**) in keratinocytes (scale bar: 100 μm). Ac_5_GalNTGc was topically applied to the dorsal skin of C57BL/6 mice: **H** Scanning electron microscopy images revealing changes in the mouse epidermis; **I** Measurement of transepidermal water loss to assess skin barrier function; **J** Observation of inflammatory manifestations on the skin and HE staining of skin tissue (scale bar: 50 μm). ^*^P < 0.05, ^**^P < 0.01^, ***^P < 0.001.

We then examined the impact of O-GalNAc glycosylation on the tight junctions of keratinocytes and skin barrier. We first synthesized the O-GalNAc inhibitor, Ac_5_GalNTGc (Fig. 4A), and employed it to treat both keratinocytes and mouse skin. We confirmed that Ac_5_GalNTGc effectively inhibited O-GalNAc glycosylation in keratinocytes (Fig. 4B, C). Subsequently, we observed that Ac_5_GalNTGc led to the disruption of tight junctions in keratinocytes (Fig. 4D, E) and decreased cellular adhesion (Fig. 4F, G). Furthermore, when Ac_5_GalNTGc was topically applied to the dorsal skin of normal mice, we noted skin barrier disruption (Fig. 4H, I), accompanied by inflammatory manifestations (Fig. 4J). Notably, the skin inflammation induced by topical Ac_5_GalNTGc could be reversed by the application of mupirocin (Fig. 4J), suggesting that the inflammation was linked to bacterial infection following the disruption of the skin barrier. Importantly, the changes induced by the O-GalNAc inhibitor in both in vitro and in vivo models were consistent with those observed in the context of SERPINB7 deficiency. Therefore, we propose that the deficiency of SERPINB7 impairs the tight junctions of keratinocytes and skin barrier by reducing O-GalNAc glycosylation.

### O-GalNAc deficiency aggravate psoriasis and AD like skin lesions

**Fig. 5:**
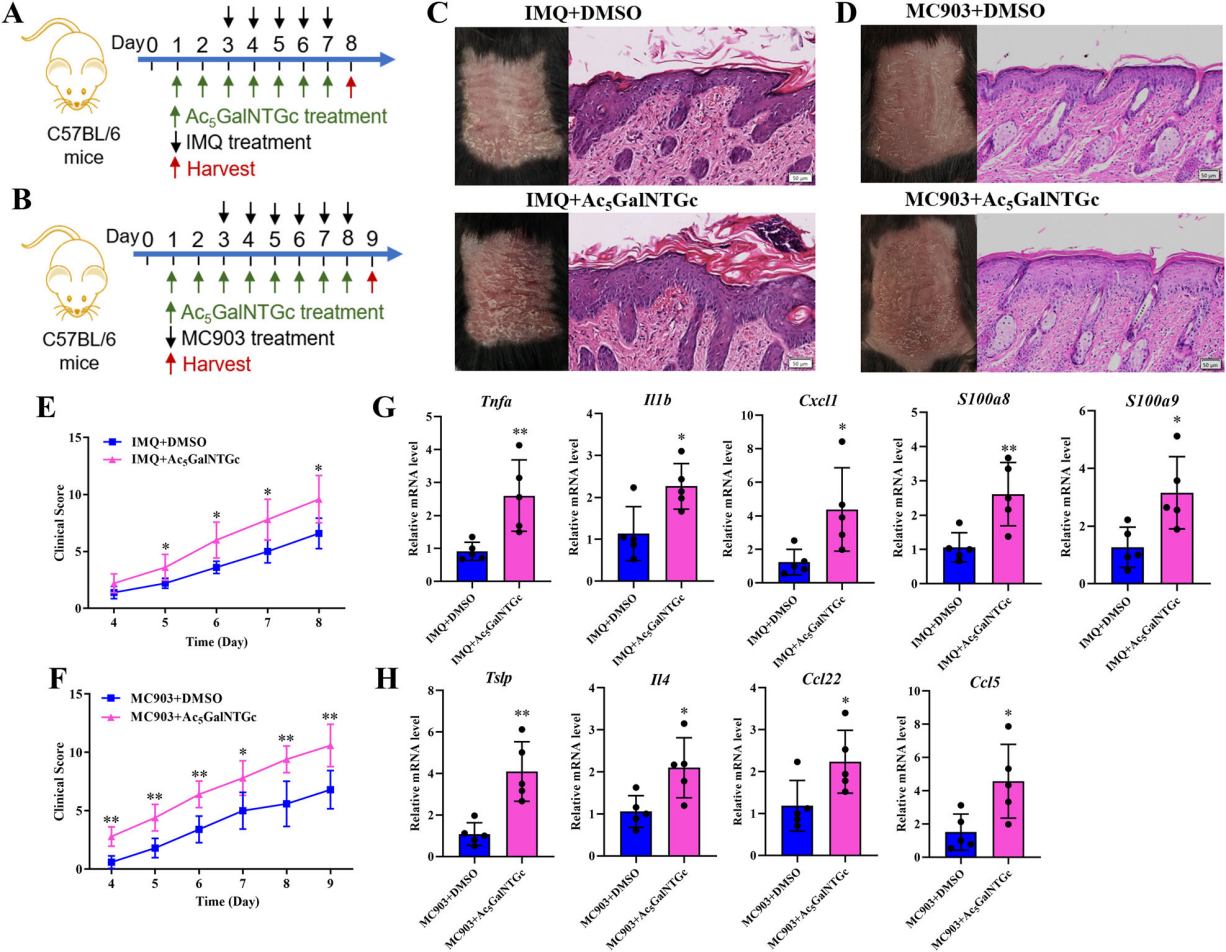
O-GalNAc deficiency aggravate psoriasis and atopic dermatitis (AD) like skin lesions. Schematic representation of the experimental design for the psoriasis (**A**) and AD (**B**) mouse models. Ac_5_GalNTGc was topically applied to the dorsal skin of the psoriasis and AD models: The macroscopic appearance and HE staining of skin tissues in psoriasis (**C**) and AD (**D**) models; Clinical scoring of skin lesion severity in psoriasis (**E**) and AD (**F**) models; qRT-PCR analysis of inflammatory factors expression in skin lesions from psoriasis (**G**) and AD (**H**) models. Scale bar, 50 μm. ^*^P < 0.05, ^**^P < 0.01, ^***^P < 0.001.

The onset and exacerbation of inflammatory skin diseases, notably psoriasis and AD, have been linked to skin barrier dysfunction. In light of this, we conducted an investigation to assess the effects of inhibiting O-GalNAc on these conditions. We developed mouse models of psoriasis and AD, induced respectively by Imiquimod (IMQ) and MC903, and applied Ac_5_GalNTGc topically to both models (Fig. 5A, B). Our results demonstrated that Ac_5_GalNTGc significantly worsened the symptoms in both psoriasis and AD models, evident through increased erythema, scaling, and lesion thickness (Fig. 5C–F). Furthermore, Ac_5_GalNTGc markedly upregulated the expression of inflammatory factors in the skin lesions of both models (Fig. 5G, H). These findings suggest that O-GalNAc deficiency exacerbates the severity of psoriasis and AD-like skin lesions.

### O-GalNAc deficiency mainly affect calcium- and adhesion-related proteins in keratinocytes

**Fig. 6:**
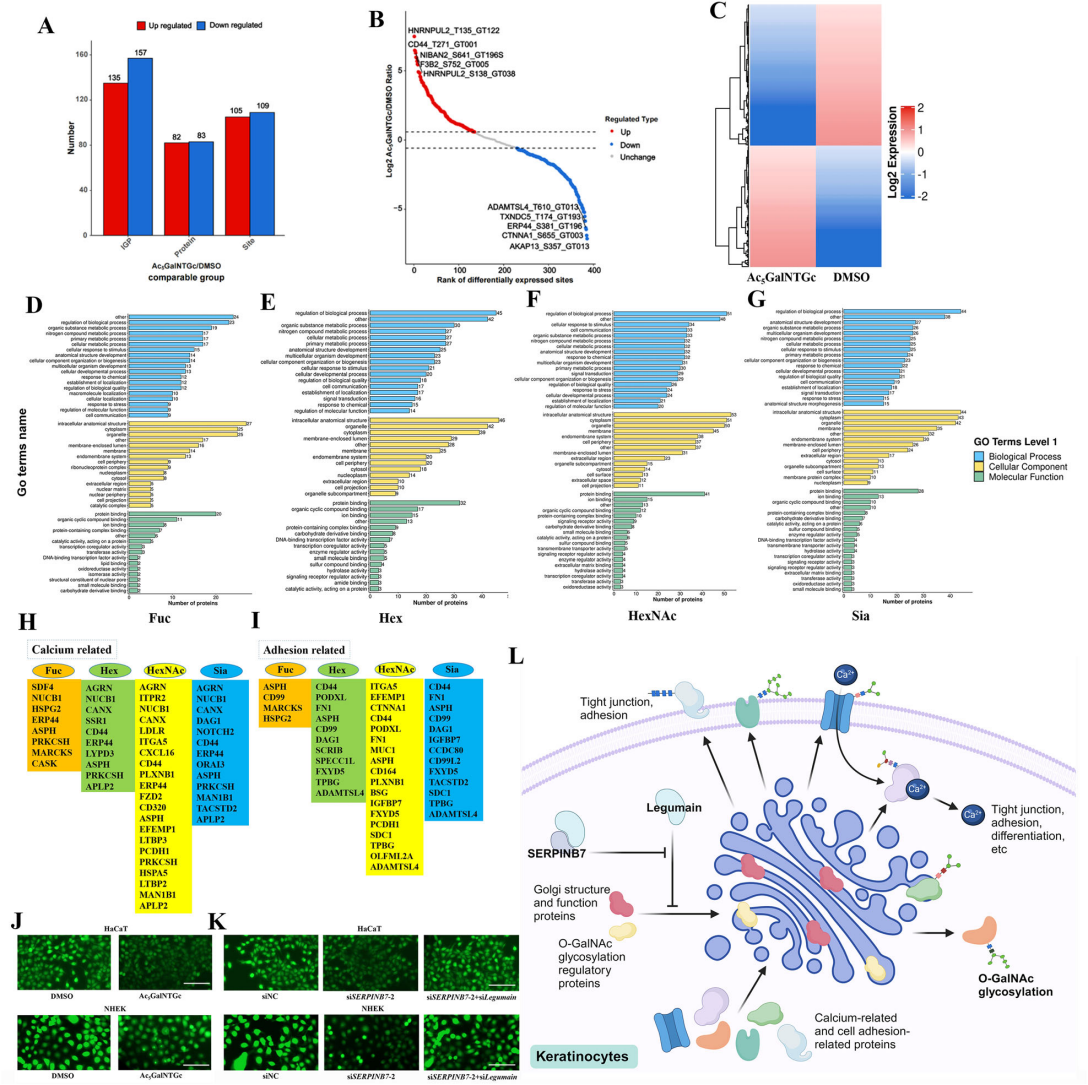
O-GalNAc deficiency primarily impacts calcium- and adhesion-related proteins in keratinocytes. Analysis of differential IGPs, their corresponding proteins, and modification sites from O-GalNAc glycopeptideomics profiling of NHEK cells treated with Ac_5_GalNTGc: statistics (**A**), scatter diagram (**B**) and heatmap (**C**) of differentially expressed IGPs. **D**–**G** GO analysis of proteins with differential modification of Fuc, Hex, HexNAc and Sia. Screening calcium-related (**H**) and adhesion-related (**I**) proteins from the differentially expressed proteins based on Fuc, Hex, HexNAc and Sia modifications. Measurement of Ca^2+^ influx changes in keratinocytes following Ac_5_GalNTGc treatment (**J**) or with SERPINB7 and Legumain knockdown (**K**) (scale bar: 100 μm). **L** Diagram showing the SERPINB7/O-GalNAc regulation in keratinocytes. ^*^P < 0.05, ^**^P < 0.01, ^***^P < 0.001.

Finally, we investigated the molecular mechanisms by which O-GalNAc deficiency affects keratinocyte biology. Through O-GalNAc glycopeptideomics analysis, we identified 292 differential intact glycopeptides (IGPs), corresponding to 165 proteins and 214 modification sites, after Ac_5_GalNTGc treatment in keratinocytes (Fig. 6A–C). Subsequent functional categorization, enrichment analysis, and clustering of these O-GalNAc-differential proteins were conducted (Fig. 6D–G, and Supplementary Figs. 4–11). These analyzes revealed several key insights. Fuc modification was predominantly found in nuclear proteins, involved in nuclear processes such as DNA binding and RNA processing (Fig. 6D–G, and Supplementary Figs. 4–11). In contrast, the other three modifications were mainly present in plasma membrane, extracellular, and endoplasmic reticulum proteins, mediating membrane-associated processes such as ion binding, transmembrane signaling, and others (Fig. 6D–G, and Supplementary Figs. 4–11).

To further elucidate the biological processes affected by O-GalNAc, we consulted the functions of these differential proteins one by one. Intriguingly, among the 165 differential proteins, 31 were associated with calcium (Fig. 6H), involving processes such as calcium binding, sequestration, and transduction. Additionally, 27 proteins were related to cell adhesion and junction (Fig. 6I). For both calcium-related and adhesion-related proteins, the most abundant modification was HexNAc, followed by Sia and Hex, with Fuc being relatively scarce (Fig. 6H, I).

Previous studies have demonstrated that SERPINB7 regulates Ca^2+^ influx [6], our findings indicate that O-GalNAc glycosylation predominantly impacts calcium-related proteins. Therefore, we further investigated whether O-GalNAc, as a downstream target of SERPINB7, could also regulate Ca^2+^ influx. Our results indicated that Ac_5_GalNTGc significantly inhibited Ca^2+^ influx in keratinocytes (Fig. 6J). Furthermore, we confirmed that the knockdown of Legumain rescued the disruption of Ca²⁺ influx induced by SERPINB7 silencing (Fig. 6K). Moreover, we had previously observed that inhibition of O-GalNAc glycosylation by Ac_5_GalNTGc caused disruption of tight junctions and decreased cell adhesion in keratinocytes (Fig. 4E–H). These findings may be related to altered O-GalNAc glycosylation of adhesion-related proteins.

Numerous studies have confirmed the roles of calcium homeostasis in keratinocyte differentiation and skin barrier, as well as the importance of cell adhesion in maintaining tight junctions and skin barrier integrity [5, 8, 9]. Incorporating previous research with our findings, we propose a mechanism where SERPINB7 deficiency alters O-GalNAc glycosylation of calcium- and adhesion-related proteins, thereby affecting their functions. These changes lead to disrupted calcium homeostasis and reduced cell adhesion, ultimately resulting in skin barrier impairment.

## Discussion

The skin barrier is a critical defense mechanism against external environmental challenges, and disruptions in this barrier are closely associated with various skin diseases, including AD and psoriasis. In this study, we confirmed that SERPINB7, a serine protease inhibitor, is essential in maintaining keratinocyte function and skin barrier integrity. Furthermore, we identified Legumain as a key mediator of these effects and demonstrated that O-GalNAc glycosylation is a downstream target of SERPINB7 in maintaining skin barrier function.

Our results demonstrate that SERPINB7 is predominantly expressed in keratinocytes, consistent with its critical role in skin barrier maintenance. We found that SERPINB7 deficiency disrupts the tight junctions of keratinocytes, thereby compromising the integrity of the skin barrier. These findings are consistent with previous studies that have linked SERPINB7 deficiency to skin barrier dysfunction, particularly in conditions such as Nagashima-type palmoplantar keratoderma and psoriasis [6, 7, 10]. Importantly, our study highlights the keratinocyte-dependent nature of SERPINB7-mediated skin barrier maintenance, filling an important knowledge gap in this biological process.

Mechanistically, this study identifies O-GalNAc glycosylation as a downstream target of SERPINB7. SERPINB7 deficiency results in the downregulation of O-GalNAc regulatory proteins and induces structural abnormalities in the Golgi apparatus, ultimately impairing protein O-GalNAc glycosylation. Notably, we identified Legumain, a cysteine protease previously established as a primary target protease for SERPINB7 [7], as a key mediator of these effects. These findings suggest that SERPINB7 regulates O-GalNAc glycosylation through its modulation of Legumain activity. Mammalian Legumain is an endolysosomal cysteine protease highly expressed in various tissues, including the kidneys, liver, spleen, placenta, and skin [11, 12]. While Legumain is primarily localized to endolysosomes, it has also been detected in the nucleus and cytosol, on cell surfaces, in the extracellular matrix, and within extracellular vesicles [12]. A large number of proteins are known to be cleaved by Legumain; cleavage of these substrates mediates processes such as the activation, processing, or more commonly, the degradation of the target proteins [12, 13]. Many studies have reported Legumain’s association with epidermal terminal differentiation and skin barrier function [11, 14]. In this study, we demonstrate that Legumain participates in the SERPINB7-dependent regulation of keratinocyte tight junctions and skin barrier maintenance. This study further substantiates the intermediary role of Legumain in SERPINB7-regulated biological processes and downstream target, thereby complementing previous research findings.

O-GalNAc glycosylation, also known as mucin-type glycosylation, is widely present on mucins at various mucosal sites, as well as on cell surface proteins and secreted proteins [15, 16]. O-GalNAc glycosylation process primarily occurs in the Golgi apparatus and is regulated by multiple GALNT enzymes [17, 18]. It plays essential roles in a wide range of physiological and pathological processes, including cell-cell communication, cell adhesion, signal transduction, immune responses, and host-pathogen interactions [16]. Previous research on O-GalNAc glycosylation has predominantly focused on the respiratory and digestive tracts, where it is critical for maintaining epithelial homeostasis and function [19, 20]. Moreover, dysregulated O-GalNAc glycosylation has been implicated in various human diseases, particularly in cancer and inflammatory conditions [17, 21–23]. Despite its importance, the role of O-GalNAc glycosylation in the skin and its contribution to skin-related diseases remain poorly understood, with its underlying mechanisms largely unexplored.

Here, we explored the role of O-GalNAc glycosylation in maintaining keratinocytes biology and skin barrier function. The effects of O-GalNAc inhibition, both in vitro and in vivo, closely resembled the phenotypic consequences of SERPINB7 deficiency, characterized by impaired tight junctions, reduced cell adhesion, and compromised skin barrier integrity in keratinocytes and mouse skin, respectively. These findings not only highlight the importance of O-GalNAc glycosylation in preserving skin barrier integrity but also establish its role as a key mediator in the skin barrier disruption caused by SERPINB7 deficiency. Furthermore, we demonstrated that O-GalNAc inhibition exacerbates inflammatory skin diseases, including psoriasis and AD. These insights provide valuable contributions to understanding the pathogenesis of psoriasis and AD and offer a foundation for developing therapeutic strategies targeting O-GalNAc glycosylation.

In this study, the O-GalNAc glycoproteome was used to identify key glycoforms involved in protein O-GalNAc glycosylation modification, including HexNAc, Sia, Hex, and Fuc. Specifically, HexNAc refers to N-acetylhexosamine and Hex refers to hexose; Fuc denotes fucose; and Sia stands for sialic acid. These glycosylation modifications act synergistically to endow proteins with diverse biological functions, which are crucial for maintaining the normal physiological activities of cells. Regarding the mechanism by which O-GalNAc glycosylation exerts its biological effects in keratinocytes, we found that O-GalNAc inhibition primarily influences calcium-related and cell adhesion-related proteins. Calcium homeostasis is critical for keratinocyte differentiation and skin barrier function, and our results demonstrate that O-GalNAc deficiency significantly reduces calcium influx in keratinocytes. This finding provides further clarification on the molecular mechanisms underlying SERPINB7-mediated regulation of Ca^2+^ influx, as reported in previous studies [6]. Additionally, the altered O-GalNAc glycosylation of adhesion-related proteins explains the disruption of tight junctions and the reduction in cell adhesion observed following both O-GalNAc inhibition and SERPINB7 deficiency. Based on these findings, we propose that SERPINB7 deficiency impairs the O-GalNAc glycosylation of calcium-related and adhesion-related proteins, leading to dysregulated calcium signaling and compromised cell adhesion, which ultimately results in skin barrier dysfunction (Fig. 6L).

This study addresses several critical gaps in the field. Firstly, it further confirms that SERPINB7 maintains skin barrier function primarily through its role in keratinocytes. Secondly, it identifies O-GalNAc glycosylation as a downstream target of SERPINB7 and demonstrates that O-GalNAc glycosylation is essential for maintaining keratinocyte tight junctions and skin barrier integrity. Thirdly, it further substantiates the intermediary role of Legumain in SERPINB7-regulated biological processes and downstream target. Furthermore, it reveals that abnormal O-GalNAc glycosylation contributes to the pathogenesis of psoriasis and atopic dermatitis. Lastly, this study clarifies that insufficient O-GalNAc glycosylation in keratinocytes primarily affects calcium-related and adhesion-related proteins, thereby disrupting calcium homeostasis and cell adhesion, ultimately impairing epidermal barrier function.

In summary, this study demonstrates that SERPINB7 deficiency impairs the O-GalNAc glycosylation of calcium- and adhesion-related proteins through its regulation of Legumain, ultimately leading to disrupted keratinocyte tight junctions and compromised skin barrier integrity. These findings not only advance our understanding of the molecular mechanisms underlying skin barrier biology but also offer novel perspectives for developing therapeutic interventions targeting skin barrier-related diseases.

## Materials and methods

More detailed methods are described in the Supplementary methods (see Supplementary Information).

### Clinical specimens

Healthy skin tissues were obtained from patients at the Department of Dermatologic Surgery of Shanghai Skin Disease Hospital. The conduct of this research was authorized by the Ethics Committee of Shanghai Skin Disease Hospital, with approval number 2024-38. All participants provided their written informed consent prior to participation.

### Cell culture, transfection and treatment

The human keratinocyte cell line, HaCaT, acquired from ATCC, was cultivated in an ideal growth medium consisting of Dulbecco’s modified Eagle’s medium fortified with 10% fetal bovine serum. NHEKs, acquired from ATCC, were maintained in DermaCult™ Keratinocyte Expansion Medium (100-0501 & 100-0502, STEMCELL, USA). All cell lines were identified by short tandem repeat analysis and tested for mycoplasma contamination. The *SERPINB7* siRNAs were synthesized by Sangon Biotech (Shanghai, China), and the siRNAs were successfully transfected into keratinocytes utilizing the Lipofectamine RNAiMAX Transfection Reagent (13778150, Invitrogen). The SERPINB7 siRNA sequences are listed as follows: siSERPINB7-1 (sense: CCUCUCUCAGAUUGAUAAGUUTT; antisense: AACUUAUCAAUCUGAGAGAGGTT); siSERPINB7-2 (sense: CGAGUUGACUUUACGAAUCAUTT; antisense: AUGAUUCGUAAAGUCAACUCGTT). Additionally, the O-GalNAc inhibitor, Ac_5_GalNTGc [24–26], was synthesized by Nafu Biological Technology (Shanghai, China), according to the previously reported synthesis method [26]. The compound was then employed to conduct experimental treatments on the keratinocytes. For cellular functional experiments, including cell adhesion assay, observation of tight junctions by electron microscopy, and immunofluorescence detection of the tight junction protein Occludin, 1.6 mM CaCl₂ was used to promote the differentiation of keratinocytes as previously described [6].

### Animal experiments

The animal study was approved by the Ethics Committee of the Shanghai Skin Disease Hospital (2024-72). The study employed female C57BL/6 mice (7–9 weeks old) and *Serpinb7* keratinocyte conditional knockout (cKO) mice for in vivo investigations. The *Serpinb7*^fl/+^ mice and KRT14-CreERT transgenic mice were provided by the Cyagen Biosciences (Suzhou, China). *Serpinb7*^fl/+^ mice were crossed to generate the *Serpinb7*^fl/fl^ mice, and the *Serpinb7*^fl/fl^ mice were crossed with the KRT14-CreERT mice to generate KRT14-CreERT; *Serpinb7*^fl/fl^ mice. The mice were administered tamoxifen (T5648, Sigma-Aldrich) intraperitoneally at a dose of 75 micrograms per gram of body weight for five consecutive days to achieve cKO of *Serpinb7* in keratinocytes.

C57BL/6 mice were randomly assigned to experimental groups using a simple randomization method, with no blinding implemented during the study. Ac_5_GalNTGc, dissolved in corn oil, was applied topically to the backs of the C57BL/6 mice to inhibit the O-GalNAc modification in the skin. Legumain inhibitor 7r (E609437, Aladdin, Shanghai, China), dissolved in corn oil, was applied topically to the backs of the cKO mice to inhibit Legumain activity in the skin. IMQ cream (5%, H20030129, Sichuan MingXin Pharmaceutical Co., LTD) was evenly applied to the backs of the mice daily for 5 days to induce a psoriasis mouse mode. MC903 (4 nM, HY-10001, MedChemExpress) was applied daily to the backs of mice to induce an AD mouse mode.

Scanning electron microscopy examination and TEWL measurement were used to assess the skin barrier function. The severity of skin lesions was evaluated using the clinical scoring for the psoriasis mouse model [27] and AD mouse model [28]. At the end of the experiment, skin samples were collected for further analysis.

### Statistical analysis

All assays were conducted a minimum of three times, with the results presented as the mean ± standard deviation (SD). The data were analyzed utilizing GraphPad Prism 8.0 software. The Kolmogorov–Smirnov test and Levene’s test were used to test the normal distribution and homogeneity of variances successively. Comparisons between two groups employed the two-tailed Student’s *t*-test, while multiple group comparisons were made using one-way ANOVA, followed by the Newman–Keuls post-hoc test. Statistical significance was set at a *P*-value less than 0.05. Power analysis was performed to determine the sample size of the animal experiments.

## Supplementary information

Supplementary data

Original images of western blot

## Data Availability

All data in the present study are available from the authors.
