# Supplementary material for: SERPINB7 maintains skin barrier by regulating protein O-GalNAc glycosylation
Source: Cell Death Discov. 2025 Dec 30;12:66. doi: 10.1038/s41420-025-02935-6 (PMC12848094; doi:10.1038/s41420-025-02935-6)
Supplement: Supplementary file 1 — Supplementary data [file 41420_2025_2935_MOESM1_ESM.docx]

**SUPPLEMENTARY INFORMATION**

The following file contains supplementary material for the paper “SERPINB7 maintains skin barrier by regulating protein O-GalNAc glycosylation”.

This file is composed of:

• Supplementary methods

• Supplementary figures and relative supplementary figure legends (11 figures)

**SUPPLEMENTARY METHODS**

**qRT-PCR**

RNA was isolated from cells or tissues utilizing TRIzol reagent (Invitrogen), followed by reverse transcription with the PrimeScript™ RT Kit (Takara). Quantitative PCR was conducted using the SYBR Green Premium Ex Taq™ reagent kit (Takara) along with specific primer sets. GAPDH was employed as the endogenous control, and data were analyzed employing the 2^-ΔΔCt^ method.

**Western blot**

Total proteins were extracted using RIPA lysis buffer, and the proteins were fractionated by SDS-PAGE and transferred onto PVDF membranes. Subsequently, the membranes were incubated with primary antibodies specific for GALNT4 (12897-1-AP, Proteintech, Wuhan, China), COSMC (19254-1-AP, Proteintech), ST3GAL2 (13620-1-AP, Proteintech), GALNT5 (abs11214, Absin, Shanghai, China). GCC2 (AP10197b, Abcepta Botech, Suzhou, China), TRIP11 (26456-1-AP, Proteintech), TMED5 (30393-1-AP, Proteintech), Beta Actin (66009-1-Ig, Proteintech). The protein bands were visualized using enhanced chemiluminescence.

**Protein O-GalNAc level analysis**

Total O-GalNAc level of proteins were analyzed by O-GalNAc Modified Glycoprotein Assay Kit (ab235629, Abcam) and dot blot assay using a O-GalNAc antibody.

The O-GalNAc Modified Glycoprotein Assay Kit utilizes a modified galactosamine precursor that is directly introduced into the cells. This precursor is processed through the GalNAc salvage pathway to produce the intermediate uridine diphospho-GalNAz. This intermediate is then recognized by GalNAc transferases within the Golgi apparatus and incorporated into the glycoprotein. Subsequently, a click reaction with an alkyne-containing dye is performed, resulting in the generation of a green fluorescent signal. Finally, the fluorescence intensity is observed under a fluorescence microscope, serving as an indication of the relative level of O-GalNAc modification.

For dot blot assay, total proteins were extracted using RIPA lysis buffer. Protein sample were directly spotted onto a nitrocellulose membrane and allowed to air-dry at room temperature for 30 minutes. The membrane was then blocked in blocking buffer for 1 hour. The membranes were incubated with the O-GalNAc antibody (MAB16476, Abnova, Taiwan) overnight at 4°C, followed by incubation with an HRP-conjugated secondary antibody for 1 hour at room temperature. The images were visualized using enhanced chemiluminescence.

**Tandem mass tag (TMT)-based proteomic analysis**

The TMT-based proteomic analysis was conducted in collaboration with Westlake Omics (Hangzhou, China). Total proteins were extracted from HaCaT cells that had been transfected with either siNC or si*SERPINB7*. These proteins were then digested to produce peptide fragments. The resulting peptide samples were labeled using TMT 16-plex reagents (Thermo Fisher). Following labeling, the samples underwent further fractionation using a 2-hour basic pH reverse phase liquid chromatography gradient facilitated by a DIONEX Ultimate 3000 UHPLC system (Thermo Fisher). For the purpose of liquid chromatography-mass spectrometry (LC-MS/MS) analysis, a combination of the Vanquish Neo UHPLC system and the Orbitrap Exploris 480 mass spectrometer (Thermo Scientific) was employed. The proteomic data generated from this analysis were analyzed using Fragpipe software (version 19.1).

Differentially expressed proteins were identified based on a significance threshold of *P*-value < 0.05 from an unpaired Student's t-test and a fold change > 1.2. These proteins were further subjected to Gene Ontology (GO) functional analysis, Kyoto Encyclopedia of Genes and Genomes (KEGG) pathway analysis, and Gene Set Enrichment Analysis (GSEA).

**Cell adhesion assay**

Cells were plated onto cell culture dishes and subjected to either transfection or Ac_5_GalNTGc treatment. Following a 48-hour incubation period, the cells were trypsinized, counted, and prepared for subsequent adhesion experiments.

For the cell-substrate adhesion assay, the cells were resuspended in culture medium and plated at a density of 5×10^4^ cells per well into a 96-well plate that had been precoated with matrix gel. The cells were then incubated in a 37°C, 5% CO_2_ incubator for 60 minutes. After incubation, the cells were gently washed three times with PBS to remove any unbound cells. Subsequently, 100 μL of culture medium and 10 μL of CCK-8 solution were added to each well, and the plates were incubated at 37°C for appropriate time. The absorbance values at 450 nm for each well were then measured to represent the relative cell-substrate adhesion capacity.

For the cell-cell adhesion assay, prior to cell collection, the cells were labeled with 5 μM of 5(6)-CFDA, SE (SB-C4070, ShareBio, Shanghai, China) for 20 minutes. The cells were resuspended in culture medium and plated at a density of 2×10^5^ cells per well into a 24-well plate that had been preseeded with the same kind of keratinocytes at 100% confluence. The cells were then incubated in a 37°C, 5% CO_2_ incubator for 2 hours to allow for cell-cell interactions to occur. After incubation, the cells were gently washed three times with PBS to remove any unbound cells. The number of adherent cells in five predefined fields of view per well was then counted using a fluorescence microscope, with a higher cell count indicating greater cell-cell adhesion capacity.

**O-GalNAc glycoproteome**

The O-GalNAc glycoproteome analysis was conducted with the assistance of PTM BIO (Hangzhou, China), following a previously reported research methodology [1]. Initially, cells were lysed to extract total proteins, which were subsequently digested with trypsin. Subsequently, the sample underwent reduction with 5 mM dithiothreitol for 30 minutes at 56°C, followed by alkylation with 11 mM iodoacetamide for 15 minutes in the dark at room temperature. Ultimately, the peptides were purified by desalting using a Strata X SPE column.

LC-MS/MS Analysis was conducted using the Vanquish Neo UPLC system in conjunction with the high-resolution mass spectrometer, Orbitrap Astral (ThermoFisher Scientific). Raw data from the analysis were processed through MSFragger software (version 3.4), where tandem mass spectra were searched against the Homo_sapiens_9606_SP_20231220.fasta (20,429 entries) concatenated with a reverse decoy database. For enzymatic specificity, strict trypsin and cleavage at the N-terminal of ST were selected, with a tolerance of 2 and 5 missed cleavages, respectively. The peptide length was restricted to a range of 7-50 amino acids. Carbamidomethyl modification on Cysteine was designated as a fixed modification, while acetylation on the protein N-terminal and oxidation on Methionine were specified as variable modifications. Mass offsets were set according to the default list of glycosylated modifications. The false discovery rate (FDR) for proteins, peptides, and peptide spectrum matches (PSMs) was adjusted to be less than 1%. The fold change (FC) was calculated as the ratio of the relative quantitative values of intact glycopeptides between two samples. A threshold of greater than 1.5-fold change was considered significant for upregulation, while a threshold of less than 1/1.5-fold change was considered significant for downregulation.

Based on the characteristics of the identified glycans, they were classified into four glycopatterns: N-acetylhexosamine (HexNAc), hexose (Hex), fucose (Fuc), and sialic acid (Sia). Comprehensive functional annotation was conducted for proteins with differential modifications associated with these four glycopatterns, covering aspects such as Gene Ontology (GO), Protein domain, KEGG pathways, COG/KOG functional classifications, subcellular localization, Reactome, WikiPathways, Hallmarks, and Transcription factors (TFs). Enrichment analysis was performed on the differentially modified proteins using Fisher's exact test, focusing on functions related to GO, KEGG, Protein domain, Reactome, and WikiPathways. Subsequently, cluster analysis was carried out to identify similarities and differences in the protein functions corresponding to the four glycopatterns within the comparison groups.

**Measurement of intracellular Ca^2+^**

Intracellular Ca^2+^ was measured using Fluo-4 Calcium Assay Kit (S1061, Beyotime Biotechnology). Cells were seeded into a 96-well plate and treated with Ac_5_GalNTGc. After a 24-hour incubation with 1.6 mM Ca^2+^, 100 μL of Fluo-4 staining solution was added to each well. The plate was then incubated at 37°C in the dark for 30 minutes. Following the incubation, the intensity of green fluorescence was observed using a fluorescence microscope.

**References**

1. Yang W, Song A, Ao M, Xu Y, Zhang H. Large-scale site-specific mapping of the O-GalNAc glycoproteome. Nat Protoc. 2020;15:2589-610.

**SUPPLEMENTARY FIGURES**


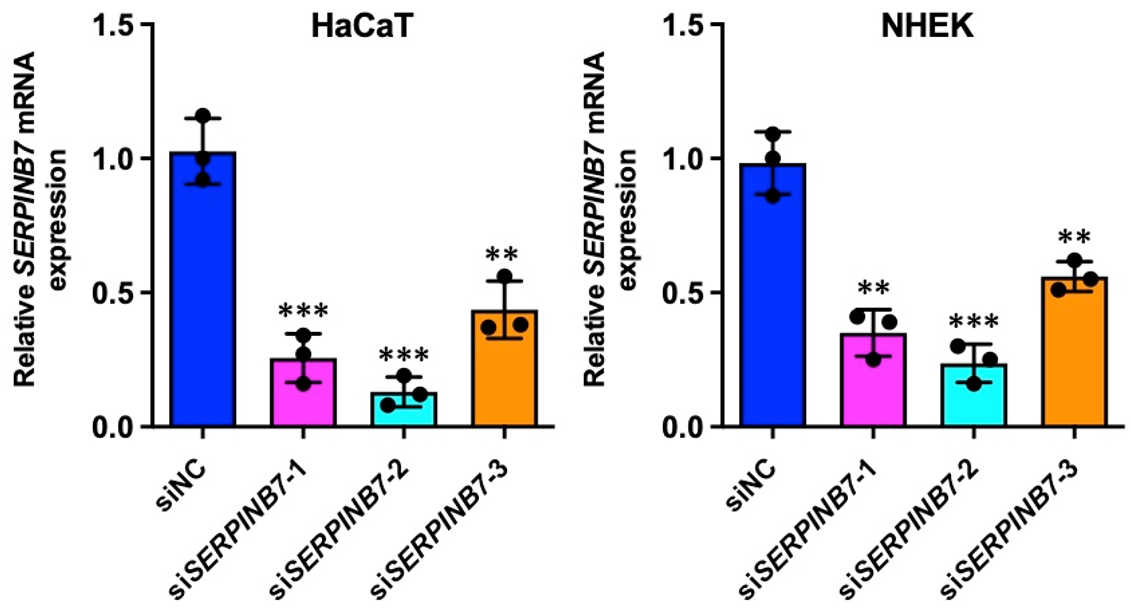
 **Supplementary Fig. 1** *SERPINB7* knockdown efficiency in keratinocytes analyzed by qRT-PCR. ^**^*P*<0.01, ^***^*P*<0.001.


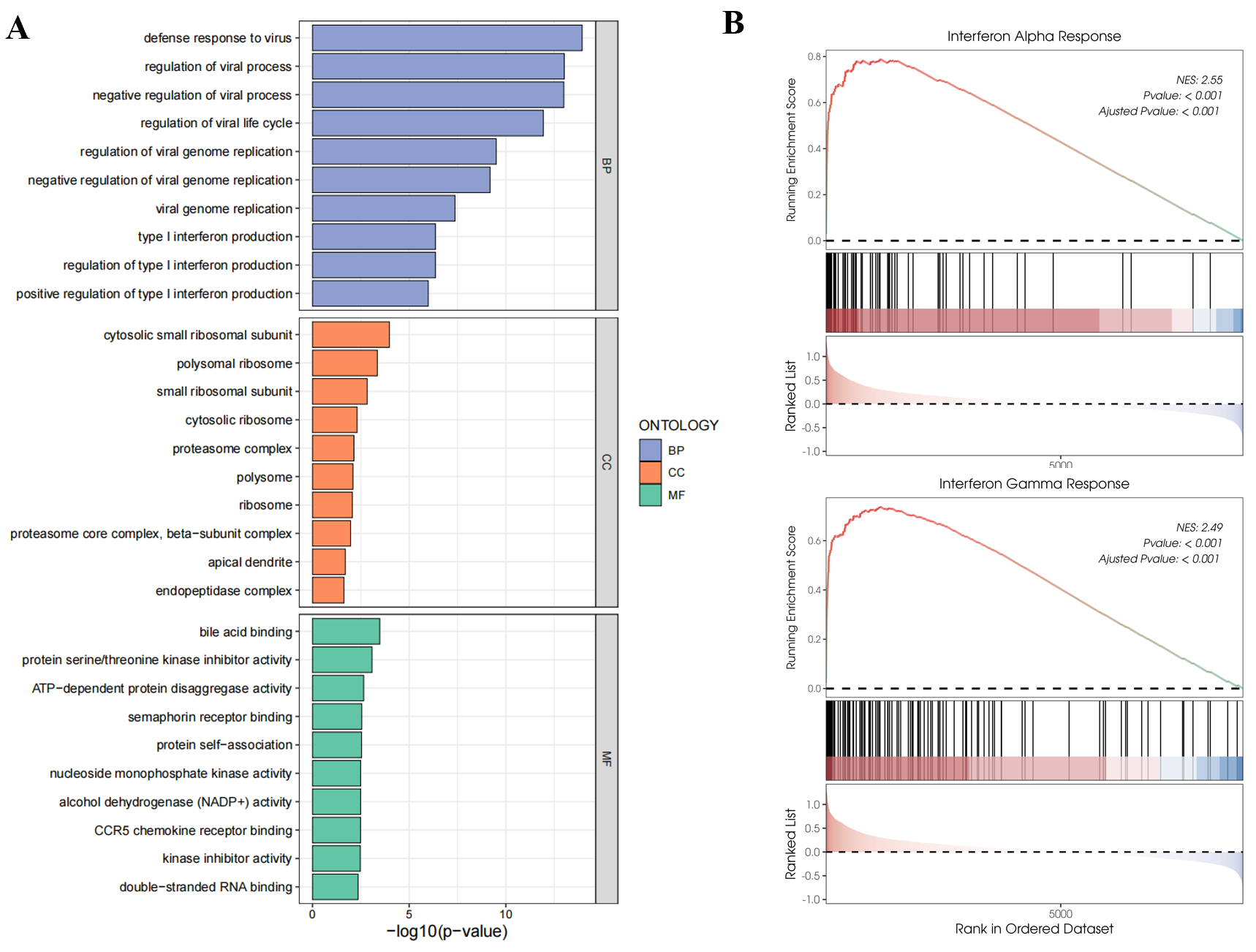
 **Supplementary Fig. 2** Gene Ontology Enrichment Analysis (A) and Gene Set Enrichment Analysis (B) of proteins upregulated following the knockdown of *SERPINB7* in keratinocytes.


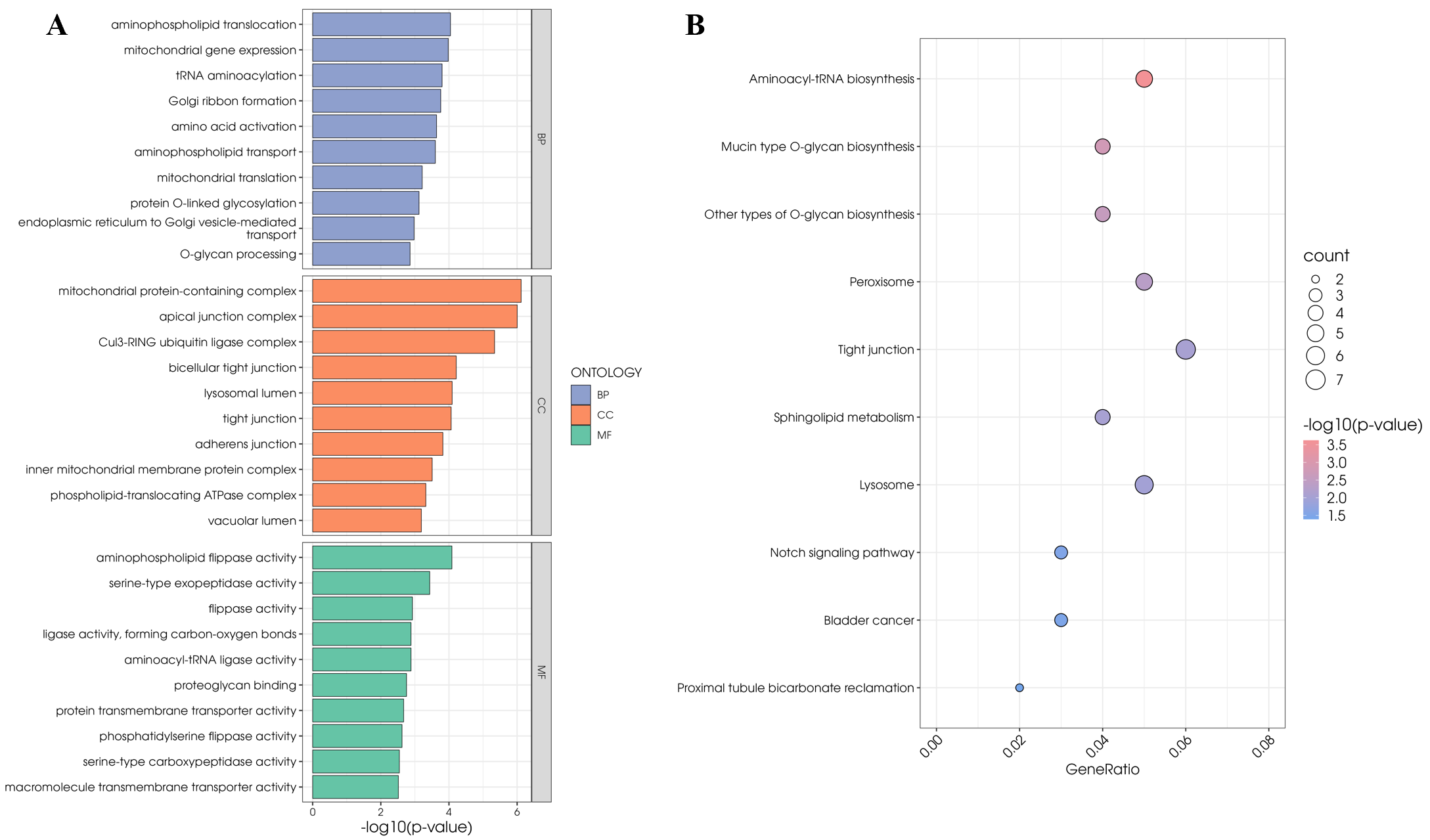
 **Supplementary Fig. 3** Gene Ontology Enrichment Analysis (A) and KEGG Enrichment Analysis (B) of proteins downregulated following the knockdown of *SERPINB7* in keratinocytes.


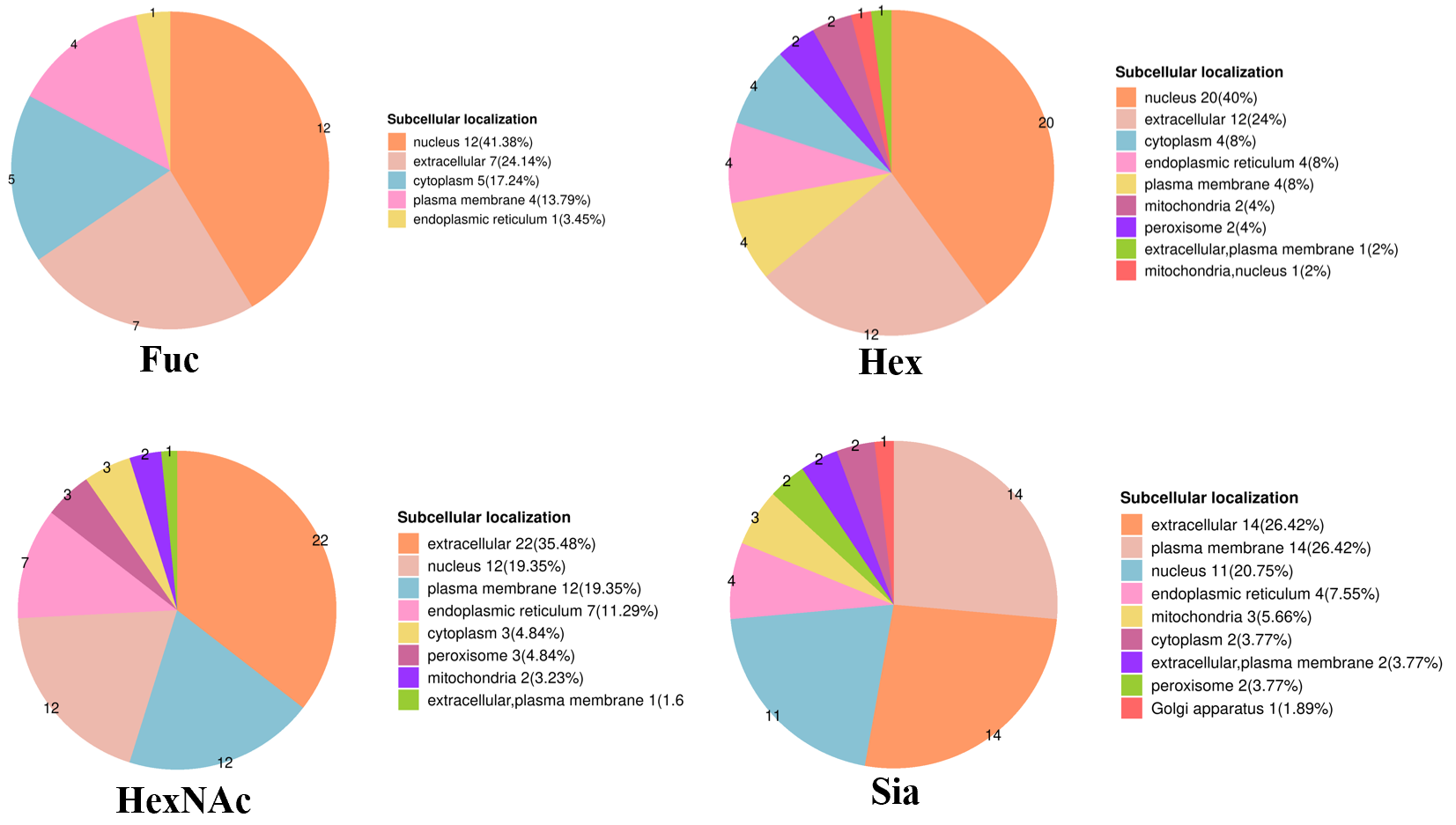
 **Supplementary Fig. 4** Subcellular localization classification of proteins with differential modification of Fuc, Hex, HexNAc and Sia in keratinocytes treated with Ac_5_GalNTGc.

**Supplementary**
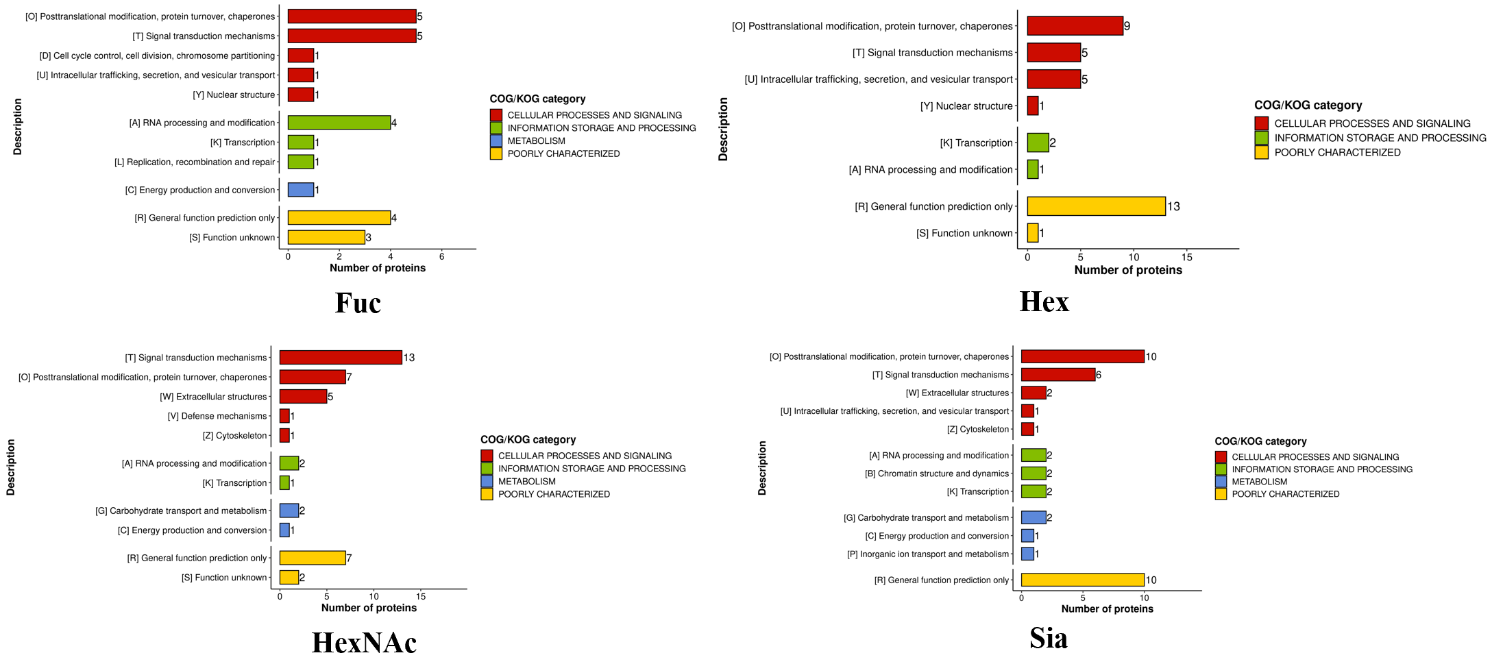
**Fig. 5** COG/KOG classification of proteins with differential modification of Fuc, Hex, HexNAc and Sia in keratinocytes treated with Ac_5_GalNTGc.


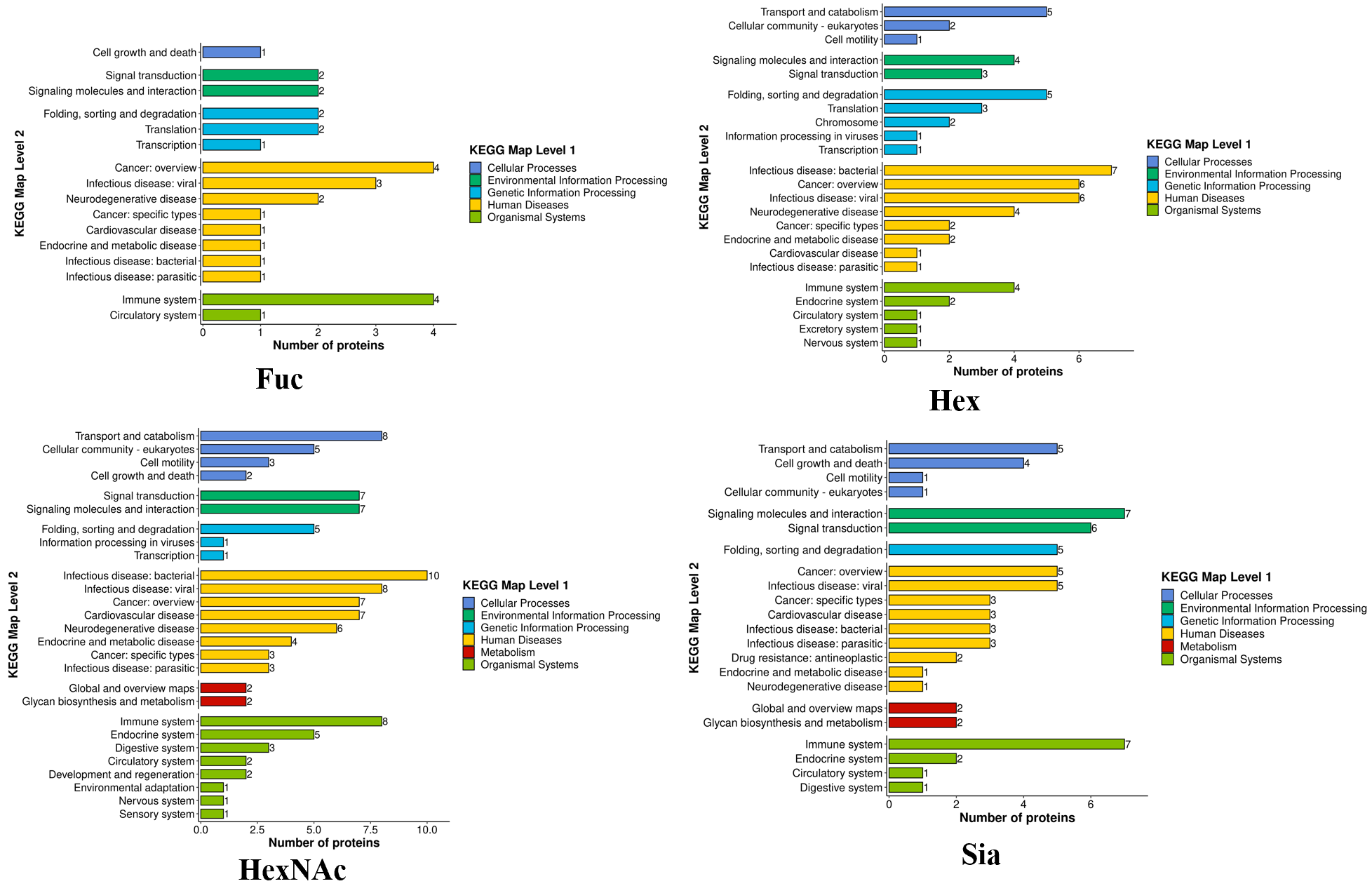
 **Supplementary Fig. 6** KEGG pathway classification of proteins with differential modification of Fuc, Hex, HexNAc and Sia in keratinocytes treated with Ac_5_GalNTGc.

**Supplementary**
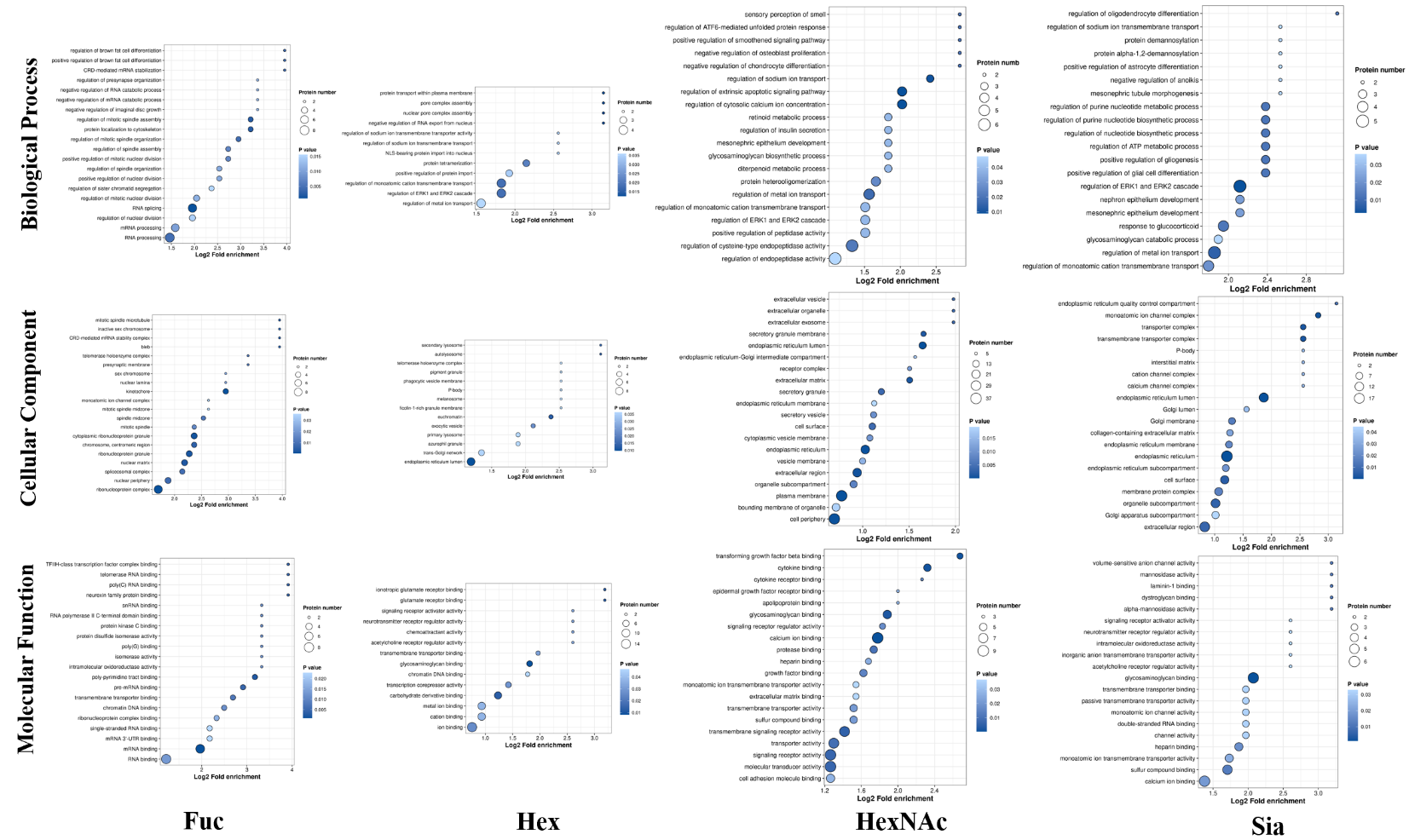
**Fig. 7** GO enrichment of proteins with differential modification of Fuc, Hex, HexNAc and Sia in keratinocytes treated with Ac_5_GalNTGc.


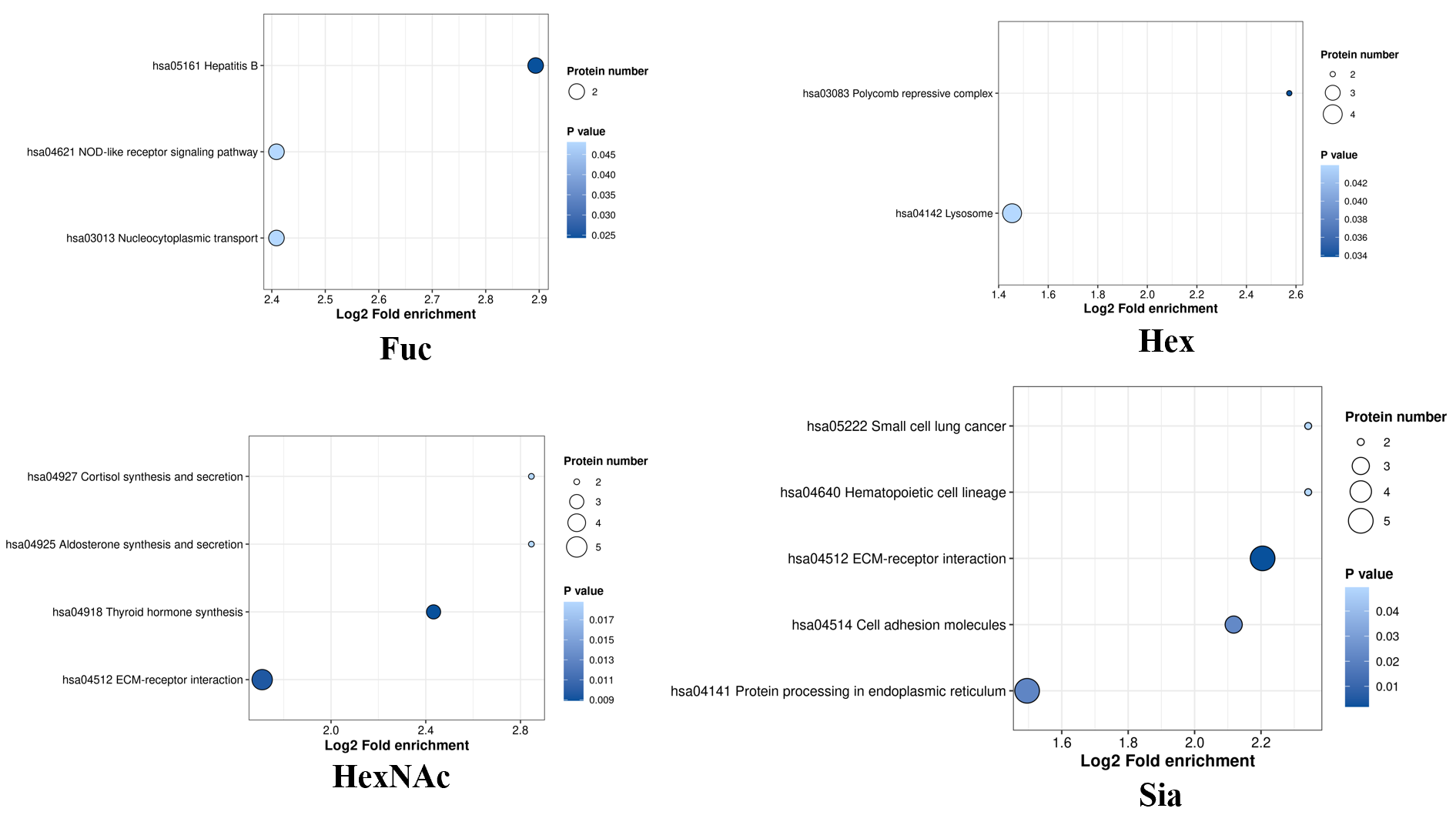
 **Supplementary** **Fig. 8** KEGG pathway enrichment of proteins with differential modification of Fuc, Hex, HexNAc and Sia in keratinocytes treated with Ac_5_GalNTGc.

**Supplementary**
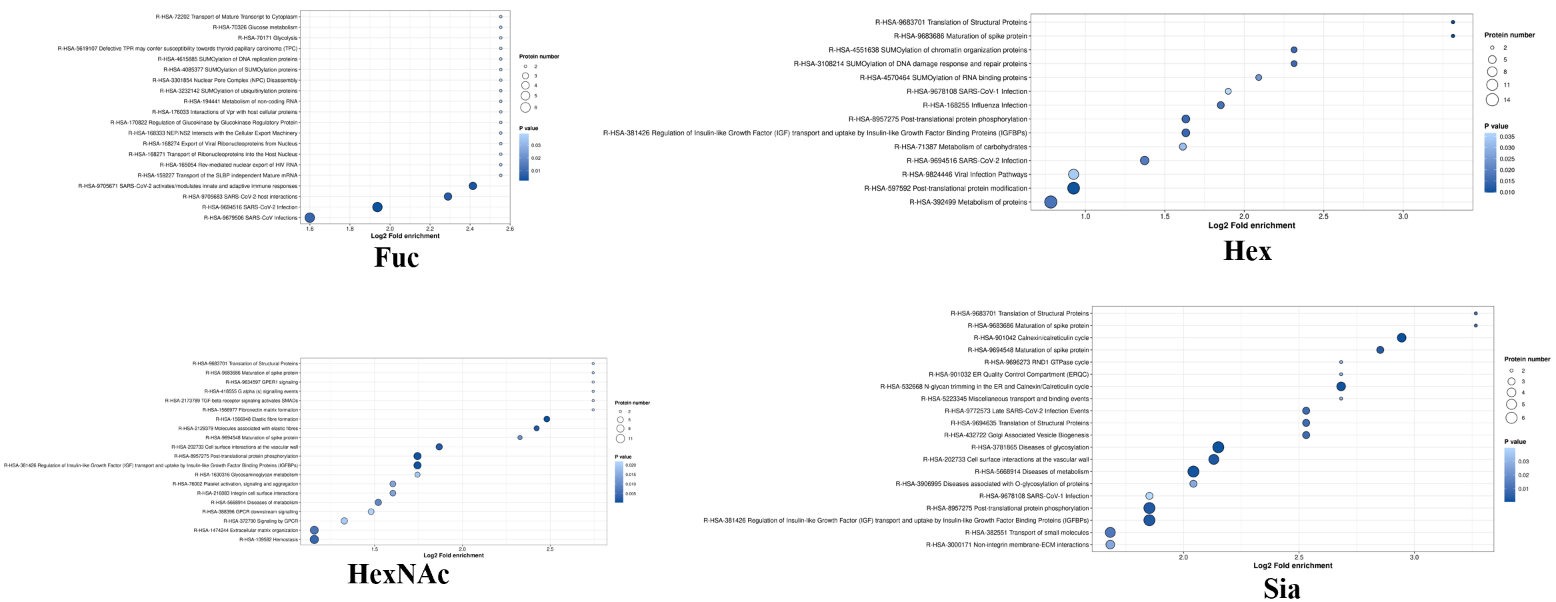
**Fig. 9** Reactome pathway enrichment of proteins with differential modification of Fuc, Hex, HexNAc and Sia in keratinocytes treated with Ac_5_GalNTGc.

**Supplementary**
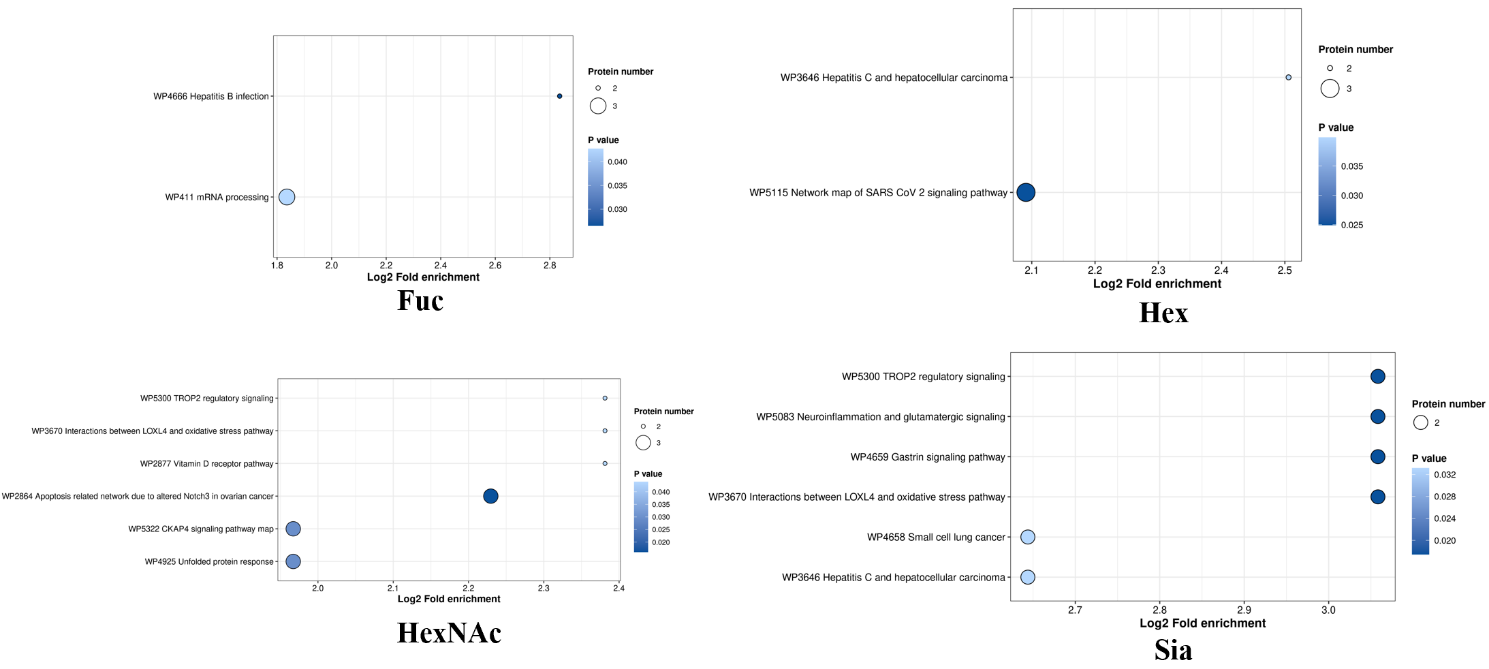
**Fig. 10** WikiPathways enrichment of proteins with differential modification of Fuc, Hex, HexNAc and Sia in keratinocytes treated with Ac_5_GalNTGc.

**Supplementary**
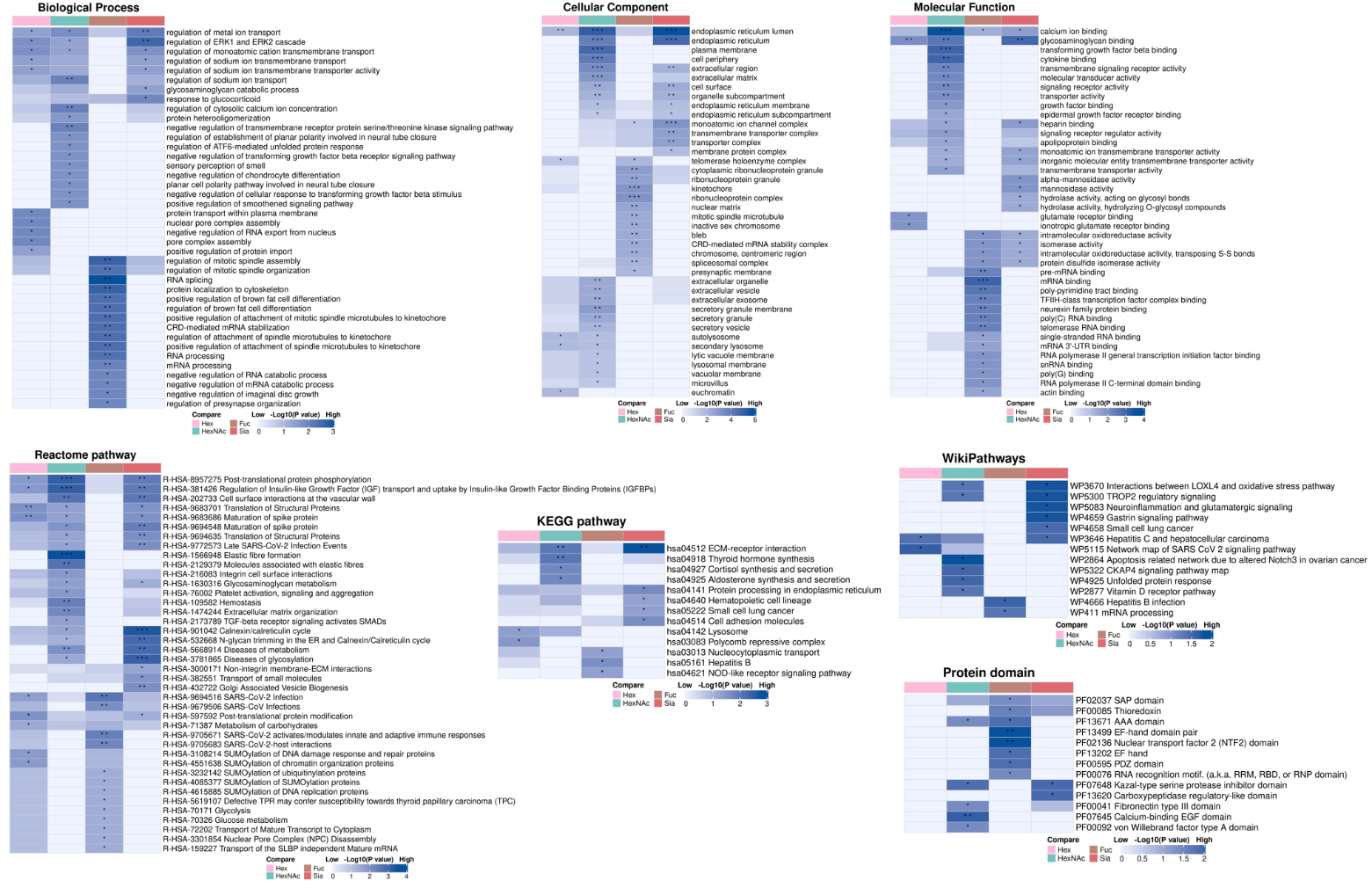
**Fig. 11** Cluster analysis of differential proteins based on glycopatterns in keratinocytes treated with Ac_5_GalNTGc. ^*^*P*<0.05, ^**^*P*<0.01, ^***^*P*<0.001.
