# Supplementary figures and images for: SERPINB7 maintains skin barrier by regulating protein O-GalNAc glycosylation
Source: Cell Death Discov. 2025 Dec 30;12:66. doi: 10.1038/s41420-025-02935-6 (PMC12848094; doi:10.1038/s41420-025-02935-6)

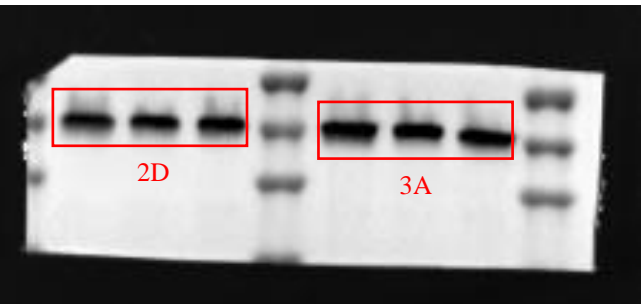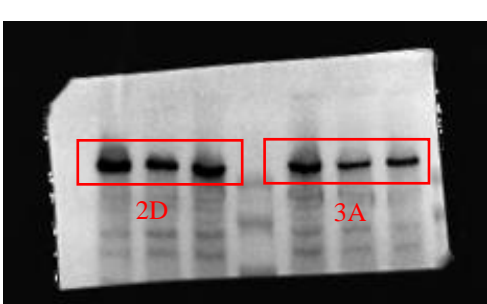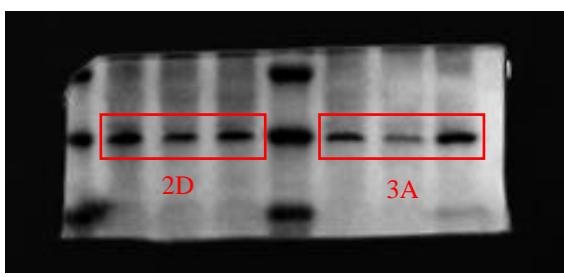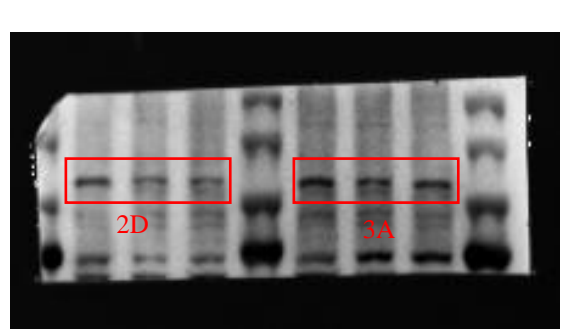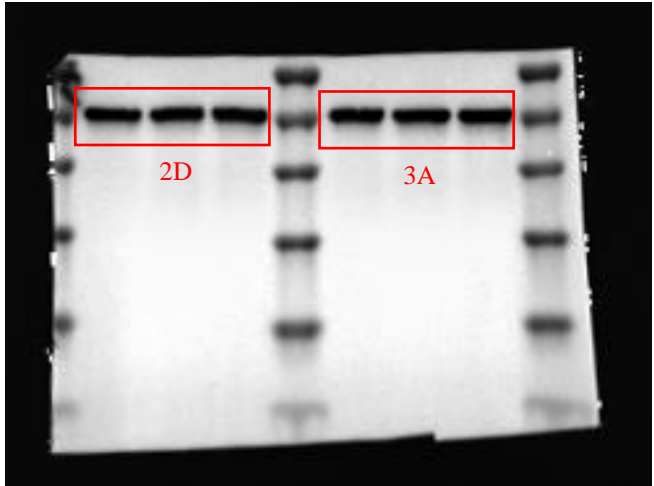

**$\beta$ -Actin**

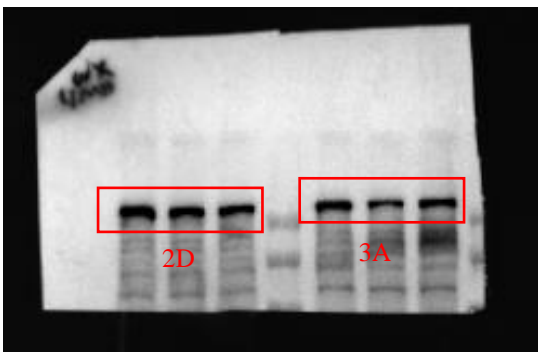

**TRIP11**

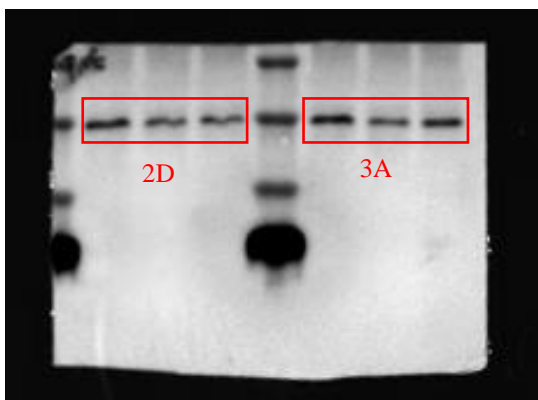

**TMED5**

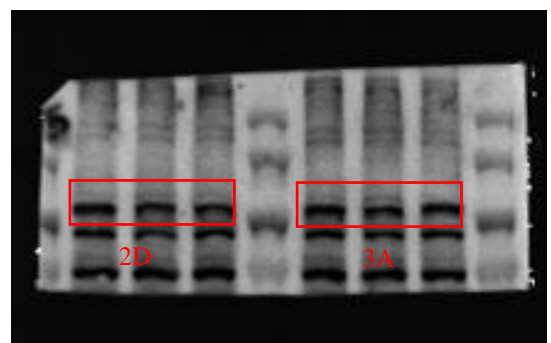

**GALNT5**

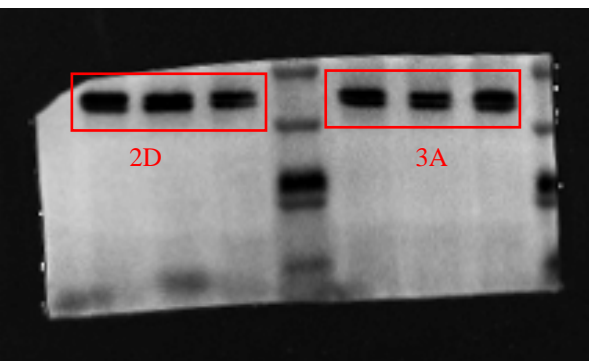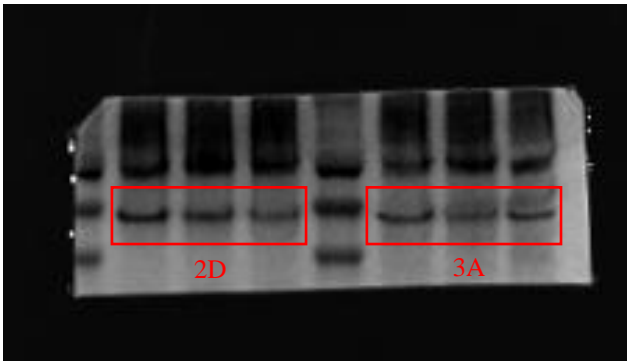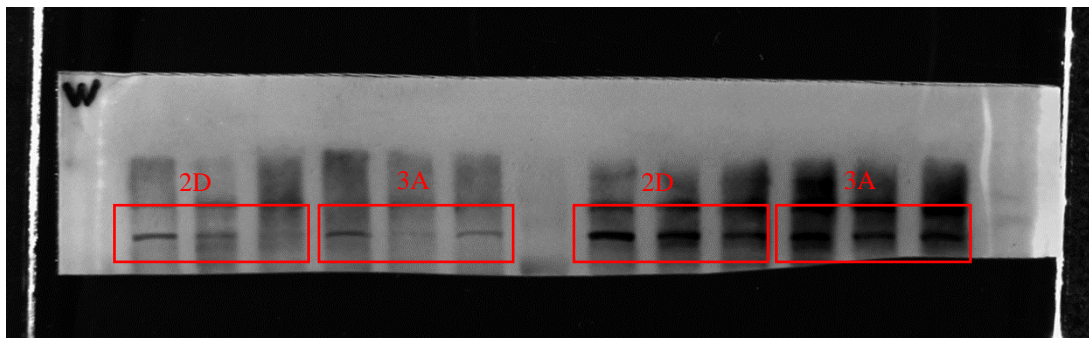

**GCC2**

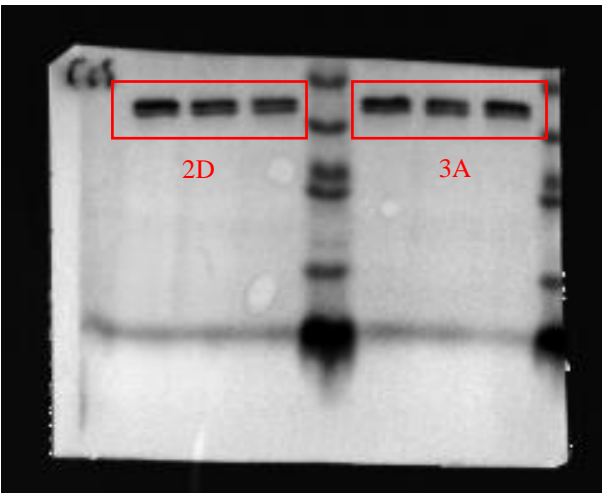

**COSMC**

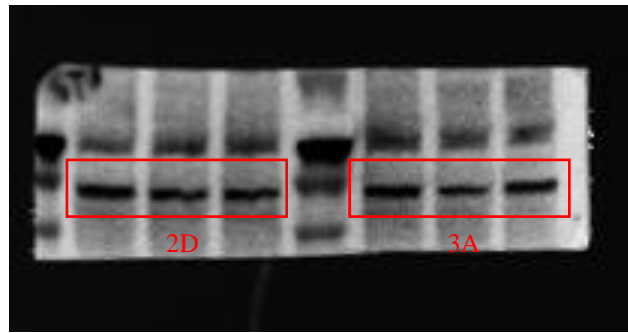

**ST3GAL2**

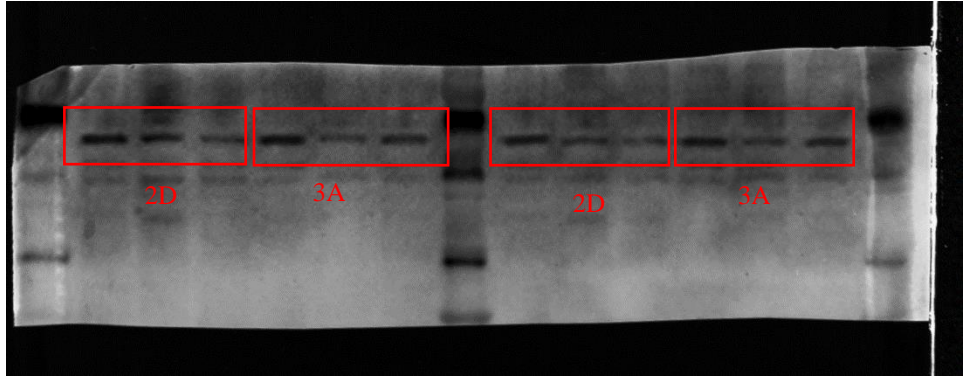

**GALNT4**

Supplement: Supplementary file 2 — Original images of western blot [file 41420_2025_2935_MOESM2_ESM.pdf]
